# A Theoretical and Experimental Study on the Critical Clamping Force for Suppressing Buckling in In-Plane Tension–Compression Testing of Sheet Metals

**DOI:** 10.3390/ma19153166

**Published:** 2026-07-23

**Authors:** Shuo Wang, Lin Zhu, Yibo Su, Chunyu Ou, Leheng Huang, Yanli Lin, Yingguang Zhao, Qi Yang, Zhubin He

**Affiliations:** 1 State Key Laboratory of High-Performance Precision Manufacturing, Dalian University of Technology, Dalian 116024, Chinasuyibo2021@163.com (Y.S.);; 2 School of Mechanical Engineering, Dalian University of Technology, Dalian 116024, China

**Keywords:** Bauschinger effect, cyclic tension–compression test, compressive instability and wrinkling, critical clamping force

## Abstract

Accurate characterization of the Bauschinger effect is essential for improving springback prediction in simulations of complex sheet-metal components. However, in-plane tension–compression tests are prone to compressive instability and wrinkling during reverse loading, and the selection of clamping force still lacks a theoretical basis. In this study, a critical clamping force prediction model was developed based on energy conservation and the Cao–Boyce instability criterion. The model establishes the relationship between the critical clamping force, material strength coefficient, strain-hardening exponent, specimen geometry, and effective support area. Finite element simulations and experiments were conducted to investigate the contact state, local support effect, and instability-mode transition of Q890 high-strength steel, 2A14 aluminum alloy, and 304 stainless steel under different clamping forces. For Q890 steel, the critical clamping force interval was 900–1000 N, within which the compressive instability strain increased from nearly 0 to 0.085 and the instability mode changed from single-wave to double-wave buckling. After calibrating the boundary correction coefficient using Q890 steel, the predicted critical clamping forces for 2A14 aluminum alloy and 304 stainless steel were 594 N and 278 N, respectively. The optimized clamping forces enabled smooth cyclic tension–compression curves.

## 1. Introduction

With the increasing requirements for lightweight structures and service safety in the aerospace, automotive, rail transportation, and other engineering fields, high-performance sheet metals, such as high-strength steels and aluminum alloys, have been increasingly used in engineering applications [1]. During complex forming processes, sheet metals often undergo multiple non-proportional loading paths and loading-path reversals, and their reverse plastic behavior affects forming limits, springback prediction, and service performance evaluation [2].

The Bauschinger effect refers to the significant reduction in yield strength of metallic materials during reverse loading after prior forward pre-deformation. This effect was first discovered and named by the German scholar J. Bauschinger [3] in 1886. In practical sheet metal stamping processes, the loading path is usually complex, and local regions of the material may experience tension–compression loading-path reversal. Under such conditions, the Bauschinger effect is important for accurately characterizing material hardening behavior and improving the predictive accuracy of forming simulations. Although the in-plane tension–compression test cannot fully reproduce the multiaxial deformation path in stamping processes, it provides reliable stress–strain data under a controlled reverse-loading condition, thereby providing a basis for accurate characterization of the Bauschinger effect and subsequent constitutive model calibration. Since classical isotropic hardening models cannot accurately describe such problems, various advanced continuum-based physical and mechanical constitutive models have been proposed to precisely capture the mechanical behavior of materials during loading reversal [4,5,6].

In-plane cyclic tension–compression tests have been widely used because they can provide uniform strain distributions and controllable stress states. However, a fundamental challenge remains during the compression stage: the specimen is highly susceptible to compressive instability and wrinkling. A systematic theoretical basis is still lacking for designing complex loading conditions, including normal constraints, to extend the forming limit and improve the formability of sheet metals. Therefore, a full understanding and prediction of plastic instability during the compression stage remain a fundamental issue.

To suppress instability, previous studies have mainly focused on two approaches: optimizing specimen geometry and applying lateral constraints. Boger et al. [7] developed a novel in-plane compression testing device, in which hydraulically controlled solid flat plates were applied on both sides of the specimen to provide constraints. Combined with an optimized specimen geometry, this design reduced the risk of instability in the unclamped region and enabled cyclic tension–compression testing of sheet metals under continuous large strain. However, the required clamping force was relatively high (8–10 kN), which may affect the stress state and cause it to deviate from ideal uniaxial compression. In addition, buckling could still occur in regions of the specimen that were not fully covered.

Kuwabara et al. [8] designed a comb-shaped/fork-shaped fixture and applied pressure to the fixture using a hydraulic mechanism. However, when used with slender specimens, this device makes it difficult to ensure axial alignment, which reduces the effective compressive strain range. Moreover, the fork-tooth structure is prone to damage during repeated use, limiting its practical applicability. Cao et al. [9] designed a wedge-type experimental fixture that achieved full coverage of the specimen, but the clamping force could not be measured. Meanwhile, friction and biaxial effects induced by the clamping force may introduce data errors, and subsequent calibration lacks a clear basis. In recent years, to address anti-buckling and friction-induced errors in tension–compression testing of thin sheets, Kopec and Kowalewski [10] designed an anti-buckling fixture suitable for large-deformation cyclic tension–compression loading of thin metal and composite sheets, and corrected the stress response by monitoring the friction force between the specimen and the supporting blocks. Kopec [11] further proposed an anti-buckling device for compression and fatigue testing of high-strength thin sheets, enabling axial compression tests of thin-sheet specimens without obvious buckling and improving the accuracy of stress responses through friction correction.

The above studies indicate that in-plane tension–compression tests can effectively characterize the Bauschinger effect, and previous analytical models have provided important foundations for buckling suppression and critical normal force estimation. Nevertheless, most existing models are based on idealized support conditions, whereas the actual support state in an in-plane tension–compression fixture is nonuniform. The effects of effective contact area, fixture-induced boundary deviation, and buckling-mode transition on the selection of critical clamping force therefore remain insufficiently understood. In practical testing, insufficient clamping force may lead to buckling, whereas excessive clamping force may increase friction and enhance the biaxial stress effect, resulting in significant errors in the measured stress–strain response. Therefore, it is essential to establish a theoretical model that can quantitatively predict the critical clamping force based on material properties.

At the level of theoretical analysis, the determination of instability and wrinkling mainly relies on three types of criteria [12]: plastic bifurcation theory, the dynamic criterion, and the energy criterion. Owing to the complexity of applying the dynamic criterion, it has been used less frequently in practical studies [13,14]. In contrast, plastic bifurcation theory and the energy criterion have become the main theoretical methods for instability analysis because of their higher computational efficiency, providing a feasible theoretical framework for the prediction model of clamping force.

Plastic bifurcation theory is an important foundation for the study of sheet-metal instability and was proposed by Hill [15] in 1958. This theory introduced the dynamic criterion into the analysis of material plastic instability, providing an important methodology for investigating wrinkling phenomena. It is applicable not only to the analysis of wrinkling bifurcation under compressive loading, but also to more complex instability behaviors such as tensile instability. Tugcu [16] further combined the Donnell–Mushtari–Vlasov shell theory with Hill’s bifurcation theory to predict the critical conditions for plastic buckling of plate and shell structures, thereby providing a theoretical basis for subsequent analyses of local wrinkling in sheet metals.

Senior [17] first proposed the energy method and established a highly accurate formula for calculating the critical compressive wrinkling stress. The core of this method is to prescribe the geometric profile of the wrinkle in advance, calculate the strain energy increment (U) generated by wrinkling and the work (V) done by external forces during plastic deformation, and then determine the instability state by comparing the relative magnitudes of (U) and (V): when (V) exceeds (U), wrinkling occurs. The energy method has provided an important theoretical tool for subsequent studies on the critical conditions of instability and wrinkling in sheet-metal deep drawing and tube bending processes. Yossifon and Tirosh [18] applied the energy method to the study of plastic instability in sheet metals during hydromechanical deep drawing. Based on the energy principle, they developed a prediction model for the critical blank-holding pressure, systematically analyzed the effects of key parameters such as sheet thickness, drawing ratio, hardening exponent, and curvature radius of the flange region on buckling behavior, and verified the validity of the theoretical model through forming experiments. Kim et al. [19] investigated the effect of plastic anisotropy on compressive instability of sheet metals and pointed out that the yield function and material directionality can significantly alter the instability response of sheet metals under compressive stress states.

Based on the energy method, Cao and Boyce [20] established the Cao–Boyce (C-B) instability criterion for flange wrinkling of thin sheets under lateral constraints. This criterion can effectively predict wrinkling behavior under complex geometries and boundary conditions, and is compatible with the large-deformation theoretical framework and various material constitutive models. Subsequently, Cao [21] further combined the C-B criterion with plastic bending theory and developed an analytical model for flange wrinkling in sheet-metal deep drawing. This model quantitatively revealed the functional relationships among the critical wrinkling stress, wrinkling wavelength, and normal pressure. The study indicated that sufficient mesh density is essential for accurately capturing the initial and post-buckling behavior of structures. Finally, comparisons with conical-cup and square-cup deep drawing experiments confirmed the good predictive accuracy of this series of methods. The C-B criterion provides a key theoretical tool for analyzing thin-sheet instability under constrained conditions and also lays a direct foundation for establishing the clamping force prediction model in this study.

Sheet wrinkling is essentially the result of the combined effects of material plastic deformation, structural geometry, and external constraints, and its critical state is not determined by a single factor. Previous studies have shown that material parameters determine the intrinsic ability of a sheet to resist compressive instability, geometric parameters affect the bending stiffness and instability sensitivity of the structure, and boundary conditions directly regulate the onset of wrinkling by altering the stress distribution, deformation path, and constraint level [22,23,24]. Therefore, systematic analysis of the effects of material parameters, geometric parameters, and boundary conditions on sheet wrinkling behavior can deepen the understanding of plastic instability in sheet metals and provide a theoretical basis for establishing prediction models of critical wrinkling conditions and for rational design of forming process parameters.

Extensive studies have shown that sheet wrinkling is closely related to material properties. Saxena and Dixit [25] combined bifurcation theory with finite element analysis and revealed the different effects of the hardening exponent and initial yield stress on flange wrinkling in square-cup and circular-cup deep drawing. By establishing buckling limit diagrams for IF steel sheets, Bayraktar et al. [26] showed that sheet wrinkling is closely related to material mechanical properties, stress ratio, and specimen geometry. Paquette and Kyriakides [27] pointed out that, during axial compression of tubes, the strain-hardening capacity of the material, namely a higher hardening exponent, can effectively delay the onset of wrinkling. However, when internal pressure is introduced to alter the stress state, the effect of the hardening exponent on the critical wrinkling condition is significantly weakened. This indicates that the influence mode and degree of the same material property on wrinkling behavior may vary under different stress states.

Kim and Son [28] further investigated the combined effects of material and geometric parameters on wrinkling. During compression, an increase in the anisotropy parameter of the material reduces the critical stress and thereby promotes wrinkling. Meanwhile, a decrease in sheet thickness (t) also significantly reduces the critical stress, and its influence may even exceed those of the hardening exponent and anisotropy parameter. Du et al. [29] emphasized that external constraints have a significant effect on suppressing wrinkling during nonuniform tension of thin sheets, and this effect becomes stronger as the plate size decreases. When the constraint condition changes, the influence of boundary conditions may even exceed that of sheet thickness as a geometric parameter. In addition, the same constraint configuration may exhibit different effects in different forming processes, such as flange wrinkling and sidewall wrinkling. Sivasankaran et al. [30] pointed out that wrinkling is jointly affected by material properties, punch and die geometries, blank geometry, blank-holding conditions, and friction/lubrication states. Won et al. [31] proposed a wrinkling prediction criterion for high-strength steel sheets by considering the critical compressive strain and geometric bending strain, and applied it to the prediction of wrinkling regions in actual stamped parts.

In summary, existing studies have been successfully applied to the analysis of instability and wrinkling in deep drawing, flange wrinkling, and tube bending. However, they have not been systematically applied to in-plane tension–compression tests to investigate the quantitative relationship between clamping force and material parameters, such as the strength coefficient (*K*) and the strain-hardening exponent (*n*). This lack of theoretical understanding has kept the optimization of clamping force in in-plane tension–compression tests at the stage of empirical trial and error, thereby affecting the accuracy and reliability of the tests.

Therefore, this study develops a theoretically guided and experimentally calibrated method for predicting the critical clamping force in in-plane tension–compression testing of sheet metals. The energy criterion and the Cao–Boyce instability criterion are combined to establish the baseline prediction model, and the functional relationship between the critical clamping force and material parameters, such as *K* and *n*, is quantitatively revealed. Compared with previous closed-form models based on idealized support conditions, the present study further introduces the effective support area and the boundary correction coefficient *η* to account for localized contact, effective support loss, frictional effects, and fixture-induced clamping deviation. Finite element simulations and experiments are then conducted to investigate the evolution of the contact state, the local support effect, and the transition of instability modes under different clamping forces. To verify the applicability of the model, three materials with significantly different mechanical properties, namely Q890 high-strength steel, 2A14 aluminum alloy, and 304 stainless steel, are selected for theoretical prediction, finite element simulation, and experimental validation. Finally, the model is applied to in-plane tension–compression tests of the three materials under different pre-strains to verify the feasibility of the critical instability clamping force prediction model. Stress–strain curves of the three materials under large-strain cyclic loading are obtained, and the Bauschinger effect of the three materials is characterized.

## 2. Theoretical Model of Critical Clamping Force Under Ideal Continuous Lateral Constraint

### 2.1. Compressive Instability and Principle of Lateral Constraint in In-Plane Tension–Compression Tests

In-plane tension–compression testing is an important experimental method for characterizing the Bauschinger effect of sheet metals. In this test, the specimen is usually subjected to pre-tension first, followed by reverse-compression loading. However, during the reverse-compression stage, the parallel section of the specimen is subjected to axial compressive stress and is prone to out-of-plane buckling. This causes abnormal fluctuations in the measured stress–strain curve and seriously interferes with the accurate characterization of the material constitutive behavior. Therefore, effective suppression of instability during the compression stage is the key to the successful implementation of this test.

**Figure 1:**
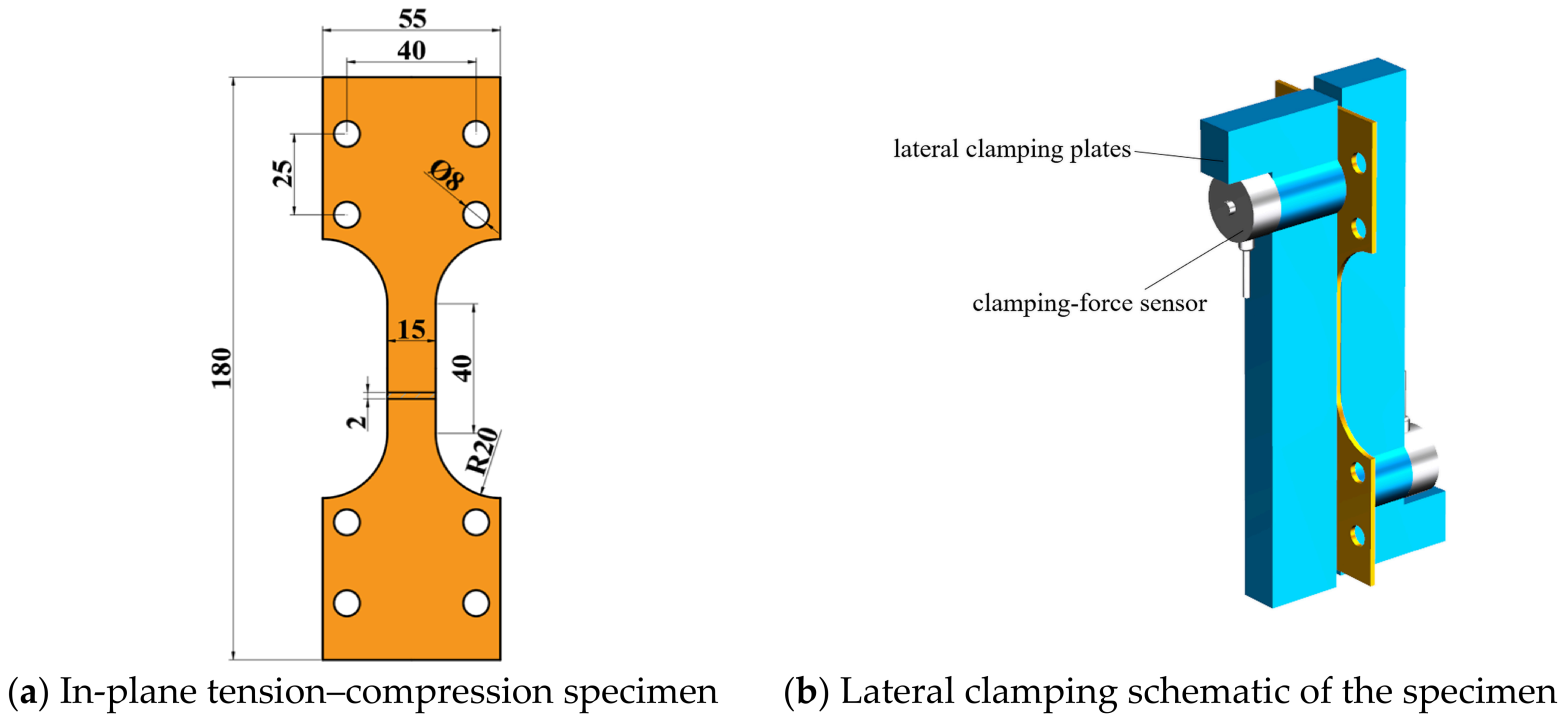
Structure of the in-plane tension–compression specimen and schematic diagram of fixture clamping.

To suppress compressive instability, lateral normal support needs to be applied on both sides of the parallel section of the specimen. Unlike existing methods that empirically determine the clamping force or provide only local constraints, the principle of the experimental device used in this study is shown in Figure 1. Its core feature is that a precisely controllable lateral clamping force is applied to provide continuous and full-coverage normal constraint over the entire parallel section of the specimen. During compression, the continuously applied clamping force can effectively suppress T-type buckling of the specimen, thereby ensuring that the mechanical response during the compression stage remains close to a uniaxial stress state.

### 2.2. Compressive Instability Analysis and Critical Clamping Force Modeling Based on the Energy Method and the Cao–Boyce Criterion

In in-plane tension–compression tests, the magnitude of the lateral clamping force has a decisive influence on the stability of the specimen during the compression stage. When the clamping force is insufficient, buckling of the specimen in the thickness direction, namely T-type buckling, cannot be suppressed. When the clamping force is excessive, significant frictional effects are introduced, causing the specimen to deviate from the ideal uniaxial stress state. Therefore, establishing a theoretical model capable of predicting the critical clamping force based on material and geometric parameters is essential for optimizing experimental conditions and ensuring testing accuracy.

**Figure 2:**
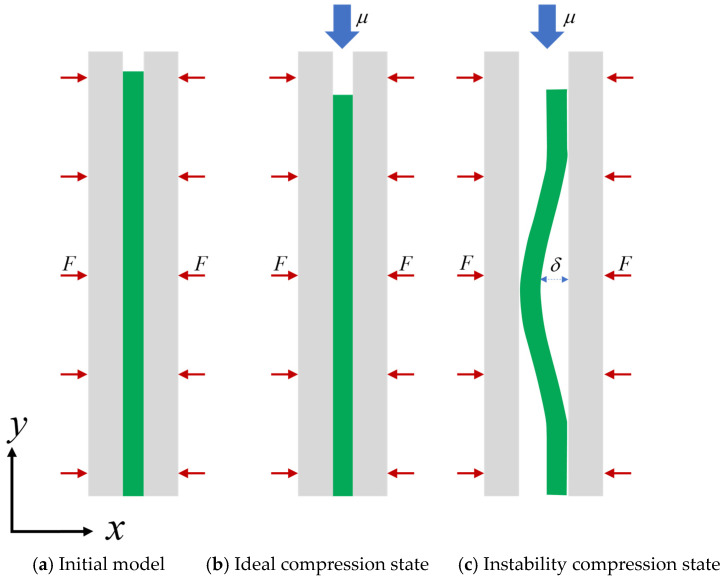
Analytical model of specimen compressive instability under ideal continuous lateral constraint.

In this study, the energy method combined with the Cao–Boyce instability criterion is used to systematically analyze and theoretically solve the compressive instability behavior of the specimen under continuous lateral constraint. To facilitate modeling, the actual boundary conditions in the experiment are reasonably simplified. Considering that compressive instability mainly occurs in the parallel section of the specimen, this region is equivalent to an ideal compressed sheet subjected to continuous normal constraints on both sides, as shown in Figure 2. The purpose of this simplification is to obtain the critical support pressure under ideal continuous constraint conditions, which serves as a theoretical baseline for the subsequent determination of the actual clamping force.

Based on the above simplification, the following basic assumptions are introduced in establishing the theoretical model:

(1) The parallel section of the specimen is regarded as an ideal sheet subjected to axial compression;
(2) The clamping plates on both sides are idealized as providing continuous and uniform equivalent normal support for establishing the baseline theoretical model;
(3) In the pre-buckling stage, the specimen thickness remains approximately unchanged, and the material satisfies the condition of volume incompressibility;
(4) Local contact nonuniformity, frictional resistance, and fixture-induced clamping deviation are not explicitly resolved in the baseline theoretical analytical model, but are subsequently considered through the effective support region and the boundary correction coefficient *η*.

Let the initial length of the specimen be $L_{s}$. When a small compressive displacement u is applied to one end, the corresponding ideal compressive strain $\varepsilon_{c}$ is given by the following expression, where compression is taken as positive, so that $\varepsilon_{c}>0$:


(1) $$\varepsilon_{c}=-\ln(1-\frac{u}{L_{s}})$$

Assume that after compressive instability occurs, the profile curve of the outer surface of the wrinkle can be described by a cosine function:

(2) $$y=\frac{\delta }{2}\left(1-\cos mx\right)$$

where *y* is the wrinkling wave function; *x* is the axial displacement of the specimen; δ is the maximum wave height in the wrinkled region; L is the wrinkling wavelength, defined as $L=L_{s}-u$; and m is the wave number of the wrinkled region, given by:


(3) $$m=\frac{2\pi }{L}=\frac{2\pi }{L_{s}\cdot e^{-\varepsilon_{c}}}$$

Based on the volume incompressibility assumption, and assuming that the specimen wall thickness remains constant during deformation, the initial length $L_{s}$ of the element before bending deformation can be expressed according to the curve arc-length formula as follows:


(4) $$L_{s}=\int_{0}^{L}\sqrt{1+{y^{\prime }}^{2}}dx\approx \int_{0}^{L}1+\frac{1}{2}{y^{\prime }}^{2}dx$$

Using Taylor expansion, the maximum instability wave height can be expressed in terms of the compressive strain as follows:


(5) $$\delta =\frac{2}{\pi }\sqrt{uL}=\frac{2L_{s}}{\pi }\sqrt{e^{-\varepsilon_{c}}-e^{-2\varepsilon_{c}}}$$

Under a given edge displacement μ, if the specimen is in an ideal compressed state without normal constraint, its plastic strain energy is denoted as $E_{0}$. When the supporting force is insufficient to suppress the instability caused by the compressive strain, the specimen buckles, and the corresponding bending plastic strain energy is denoted as $E_{W}$. Under the same edge displacement μ, the strain energy of the deformed body in the buckled state is always lower than that in the ideal compressed state. The difference between the two represents the external work required to suppress buckling:


(6) $$W_{p}=E_{0}-E_{w}$$

Let the width of the specimen parallel section be *w*, and the normal supporting force be *F*. The work $W_{p}$ done by the external force can then be expressed as follows:


(7) $$W_{p}=\int_{0}^{\delta }w\cdot Fdu$$

Following Cao and Boyce, the external work done by the normal force to suppress buckling can be expressed as:


(8) $$F=F_{max}-F_{max}{\left(\sqrt{\delta }\;-\;1\right)}^{2}$$

Thus, the critical instability pressure $p_{wr}$ can be expressed as:


(9) $$p_{wr}=\frac{F}{Lw}$$

Substituting Equation (8) into Equation (7) gives:


(10) $$W_{p}=p_{wr}\cdot \frac{2\delta L_{s}}{3}$$

Therefore, the critical pressure for suppressing instability can be obtained as:


(11) $$p_{wr}=\frac{F}{Lw}=\frac{3\left(E_{0}-E_{w}\right)}{2\delta L_{s}}$$

Under the triaxial strain state, according to the condition of volume incompressibility, the strain components satisfy $\varepsilon_{y}=-\frac{1}{2}\varepsilon_{x}=-\frac{1}{2}\varepsilon_{z}$. Thus, the equivalent strain $\bar{\varepsilon }$ can be expressed as:

(12) $$\bar{\varepsilon }=\sqrt{\frac{2}{3}\left[{\left(\varepsilon_{x}\right)}^{2}+{\left(\varepsilon_{y}\right)}^{2}+{\left(\varepsilon_{z}\right)}^{2}\right]}=\varepsilon_{z}$$

where $\varepsilon_{x}$ is the thickness-direction strain, $\varepsilon_{y}$ is the axial strain, and $\varepsilon_{z}$ is the width-direction strain.

The strain-hardening behavior of the material is described using a power-law function, where *K* is the strength coefficient and *n* is the strain-hardening exponent:


(13) $$\bar{\sigma }=K\cdot {\bar{\varepsilon }}^{n}$$

Since the volume of the ideal compressed specimen remains unchanged before and after compression and is equal to $L_{s}t_{0}$, the plastic strain energy per unit width in the ideal compression test can be expressed as:

(14) $$E_{0}=∭\bar{\sigma }d\bar{\varepsilon }dV=\frac{KL_{s}t_{0}}{n+1}{\left(\varepsilon_{c}\right)}^{n+1}$$

where

(15) $$E_{w}=\iint (\int_{0}^{\varepsilon_{w}}\sigma_{w}d\varepsilon_{w})d\rho ds=\frac{Kc^{n+1}}{n+1}{\iint \left(\left|\ln\frac{\rho }{\rho_{u}}\right|\right)}^{n+1}d\rho ds$$

where $\rho_{u}$ is the radius of curvature of the geometric neutral layer of the buckled wrinkle, and *s* is the wrinkle arc length. After derivation, Equation (15) can be transformed into:


(16) $$E_{w}=\frac{2Kt_{0}}{n+1}(\frac{t_{0}}{\sqrt{3}}{)}^{n+1}{\left(\frac{2}{m^{2}\delta }+\frac{t_{0}}{2}\right)}^{-n}\mathrm{atan}\left(\frac{m\delta }{2}\right)$$

Substituting Equations (13) and (16) into Equation (10), and rearranging, $p_{wr}$ can be obtained as:


(17) $$p_{wr}=\frac{3Kt_{0}}{2\delta (n+1)}\left({\left(\varepsilon_{c}\right)}^{n+1}-\frac{2}{L_{S}}(\frac{t_{0}}{\sqrt{3}}{)}^{n+1}{\left(\frac{2}{m^{2}\delta }+\frac{t_{0}}{2}\right)}^{-n}\mathrm{atan}\left(\frac{m\delta }{2}\right)\right)$$

Assume that the initial length $L_{s}$ of the element is a multiple of the wall thickness $t_{0}$, namely $L_{s}=\lambda t_{0}$, where λ is the normalized length and can take any real value. Substituting Equation (5) into Equation (17) and rearranging gives:


(18) $$p_{wr}=\frac{3K\pi {\left(e^{-\varepsilon_{c}}-e^{-2\varepsilon_{c}}\right)}^{-1/2}}{4\lambda (n+1)}\left({\left(\varepsilon_{c}\right)}^{n+1}-\frac{2}{\lambda 3^{(n+1)/2}}{\left(\frac{\lambda }{4\pi \sqrt{e^{3\varepsilon_{c}}-e^{2\varepsilon_{c}}}}+\frac{1}{2}\right)}^{-n}\mathrm{atan}\left(2\sqrt{e^{\varepsilon_{c}}-1}\right)\right)$$

Finally, the critical clamping force $F_{w}$ required to clamp the specimen can be expressed as:


(19) $$F_{w}=p_{wr}wL_{s}$$

Substituting Equation (18) into Equation (19) gives:


(20) $$F_{w}=\frac{3K\pi wL_{s}{\left(e^{-\varepsilon_{c}}-e^{-2\varepsilon_{c}}\right)}^{-1/2}}{4\lambda (n+1)}\left({\left(\varepsilon_{c}\right)}^{n+1}-\frac{2}{\lambda 3^{(n+1)/2}}{\left(\frac{\lambda }{4\pi \sqrt{e^{3\varepsilon_{c}}-e^{2\varepsilon_{c}}}}+\frac{1}{2}\right)}^{-n}\mathrm{atan}\left(2\sqrt{e^{\varepsilon_{c}}-1}\right)\right)$$

The above analysis indicates that the critical clamping force essentially depends on the material strength coefficient *K*, strain-hardening exponent n, specimen geometric parameters, including the normalized length λ, width w, thickness *t*, and length $L_{s}$, as well as the effective contact area.

It should be noted that the model established in this section is based on the assumption of ideal continuous lateral constraint, namely that the specimen is subjected to completely uniform normal support. Therefore, the obtained value represents the theoretical critical support-pressure baseline under uniform support conditions. However, in actual experiments, due to the combined effects of friction, clamping deviation, and thickness-direction deformation, the contact between the specimen and the clamping plates exhibits obvious localized and nonuniform characteristics, and the clamping force is not uniformly applied over the parallel section. Therefore, the results given by this theoretical model only represent the critical state under ideal continuous support conditions. The actual required clamping force should further consider the effective support region formed between the specimen and the clamping plates and the actual support pressure that can be transmitted through this region. In the following sections, this difference will be quantitatively analyzed by combining finite element simulations and experiments, and a correction method will be introduced.

### 2.3. Mode Transition and Critical Pressure Range of Compressive Instability Under Lateral Constraint

Under ideal continuous lateral constraint, compressive instability of sheet metals does not correspond to a unique instability mode. Cao and Boyce indicated that, as the normal constraint pressure increases, the wave number of the wrinkling mode gradually increases, while the corresponding wavelength and wave height decrease. When the wave number increases to a certain level, wrinkling can be regarded as being completely suppressed. This tendency indicates that the minimum critical support pressure required for different instability modes varies and changes with compressive strain.

**Figure 3:**
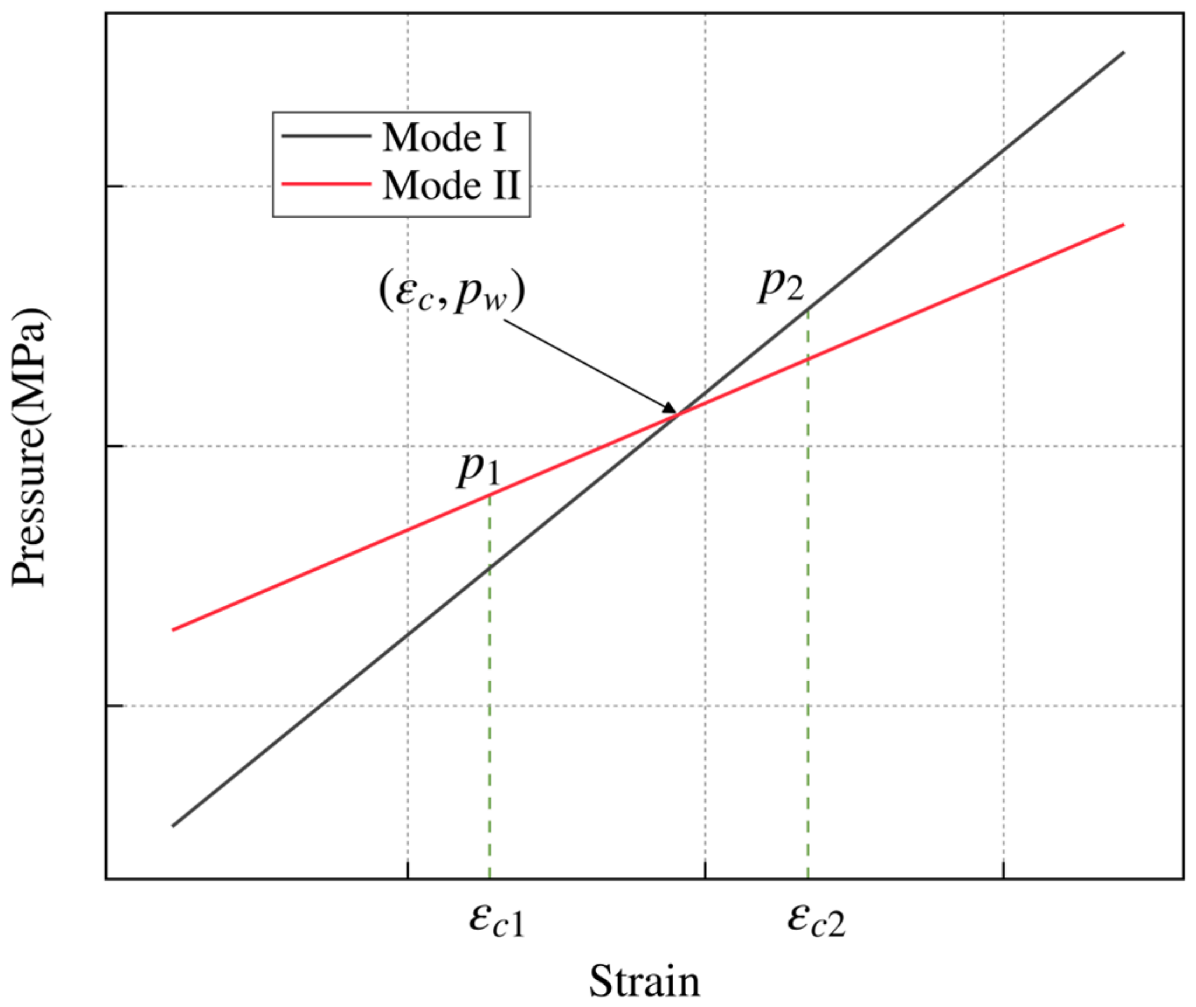
Schematic diagram of critical support pressure and mode transition for different instability modes.

Figure 3 presents the critical pressure curves corresponding to two typical instability modes, namely Mode I and Mode II, under different compressive strains. Assume that the wavelength and critical pressure of Mode I are $L_{s1}$ and $p_{1}$, respectively, while those of Mode II are $L_{s2}$ and $p_{2}$, respectively. The two curves intersect at $\varepsilon_{c}$. When the compressive strain is smaller than $\varepsilon_{c}$, the critical pressure required for Mode I is higher than that required for Mode II, indicating that a larger pressure is needed to suppress Mode I. In this case, wrinkling will preferentially occur in the form of Mode I or a lower-order mode, and $p_{1}$ should be used as the design basis in the experiment. When the compressive strain is greater than $\varepsilon_{c}$, the critical pressure required for Mode II becomes higher, and the specimen is more likely to become unstable in the form of Mode II or a higher-order mode after sufficient compression. In this case, $p_{2}$ should be used to design the clamping force. The intersection point $\varepsilon_{c}$ represents the theoretical critical strain for mode transition.

It should be emphasized that the above conclusions are based on the assumption of ideal continuous lateral constraint, namely that the specimen is subjected to completely uniform normal support, without friction or initial defects. Within this framework, mode transition appears as a well-defined critical point. However, in actual in-plane tension–compression tests, due to the combined effects of localized nonuniform contact, friction, clamping deviation, and initial geometric imperfections of the specimen, mode transition does not occur at a single theoretically predicted critical point. Instead, as the clamping force is gradually increased, the compressive stability exhibits a staged increase, accompanied by a transition of the instability mode from a lower-order mode, such as a single-wave mode, to a higher-order mode, such as a double-wave or multi-wave mode. This transition often corresponds to a relatively narrow clamping force interval rather than an isolated point.

In the following sections, this phenomenon will be quantitatively analyzed by combining finite element analysis and experimental results, and the deviation between the actual mode transition and the theoretical intersection point will be revealed, thereby providing a basis for the subsequent development of the boundary correction model.

## 3. Experimental Materials and Testing Methods

### 3.1. Experimental Materials and Characterization of Mechanical Properties

Three typical metal sheets were selected as the research materials in this study: Q890 high-strength steel, 2A14 aluminum alloy, and 304 stainless steel, all with a thickness of 2 mm. The gauge section of the uniaxial tensile specimen had uniform dimensions of 30 mm in length, 15 mm in width, and 2 mm in thickness. For the in-plane tension–compression specimen, fixed clamping regions were added at both ends based on the design of the effective deformation region in the middle of the uniaxial tensile specimen. The gauge section had dimensions of 30 mm × 15 mm × 2 mm, the parallel section length was 40 mm, and the transition filet radius was 20 mm. The total specimen length was 180 mm, the clamping region width was 55 mm, the through-hole diameter was 8 mm, and the hole spacing was 25 mm, to satisfy the installation and positioning requirements.

**Figure 4:**
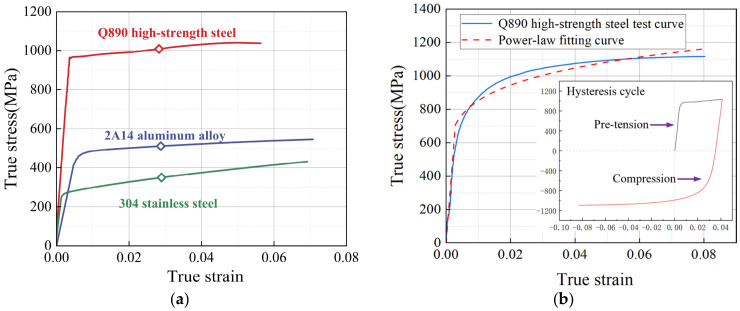

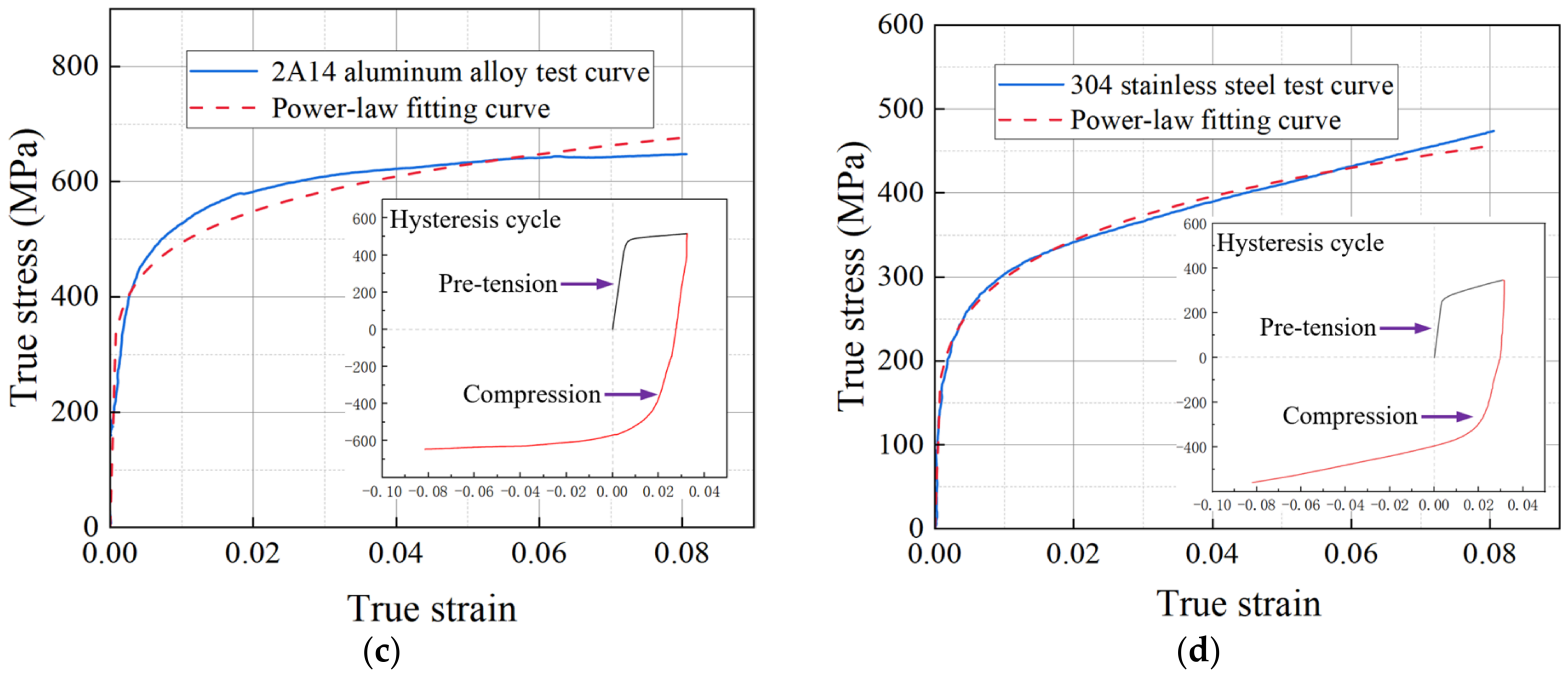
True stress–strain curves of the materials. (**a**) True stress–strain curves of the three materials under uniaxial tension. (**b**) True stress–strain curve of Q890 high-strength steel during the compression stage after pre-tension. (**c**) True stress–strain curve of 2A14 aluminum alloy during the compression stage after pre-tension. (**d**) True stress–strain curve of 304 stainless steel during the compression stage after pre-tension.

The obtained true stress–strain curves are shown in Figure 4a, and these results were mainly used to characterize the basic mechanical properties of the materials. In the in-plane tension–compression test, the specimen enters the reverse-compression stage after pre-tension, and its mechanical response is essentially different from that under uniaxial tension. Owing to the change in loading path, the compression stage usually exhibits obvious transient softening characteristics, and its yielding behavior and work-hardening law differ from those under uniaxial tension. Therefore, the strength coefficient *K* and strain-hardening exponent *n* used in the theoretical model were not obtained from the uniaxial tensile curves. Instead, they were determined by fitting the corrected true stress–strain curves in the reverse-compression stage after pre-tension using the power-law hardening model $\bar{\sigma }=K\cdot {\bar{\varepsilon }}^{n}$. The reverse-compression curves used for fitting were obtained under a specified lateral clamping force condition (2.5 kN). Before fitting, the stress contribution induced by lateral clamping was corrected by comparing the unclamped uniaxial tensile response with the tensile response measured under the same clamping condition. The obtained correction was then applied to the reverse-compression curves to reduce the influence of friction and biaxial pressure [32]. The corresponding true stress–strain curves in the compression stage are shown in Figure 4b–d.

The elastic modulus E, Poisson’s ratio ν, yield strength $\sigma_{s}$, strength coefficient *K*, and hardening exponent *n* of Q890 high-strength steel, 2A14 aluminum alloy and 304 stainless steel during the compression stage after pre-tension are listed in Table 1. Among these parameters, both *K* and *n* were obtained by fitting the true stress–strain data in the compression stage after pre-tension. It can be seen that the *K* and *n* values of the three materials differ significantly, with a maximum difference of 114.3% in *K* and 46.6% in *n*.

### 3.2. In-Plane Tension–Compression Test Setup and Procedure

**Figure 5:**
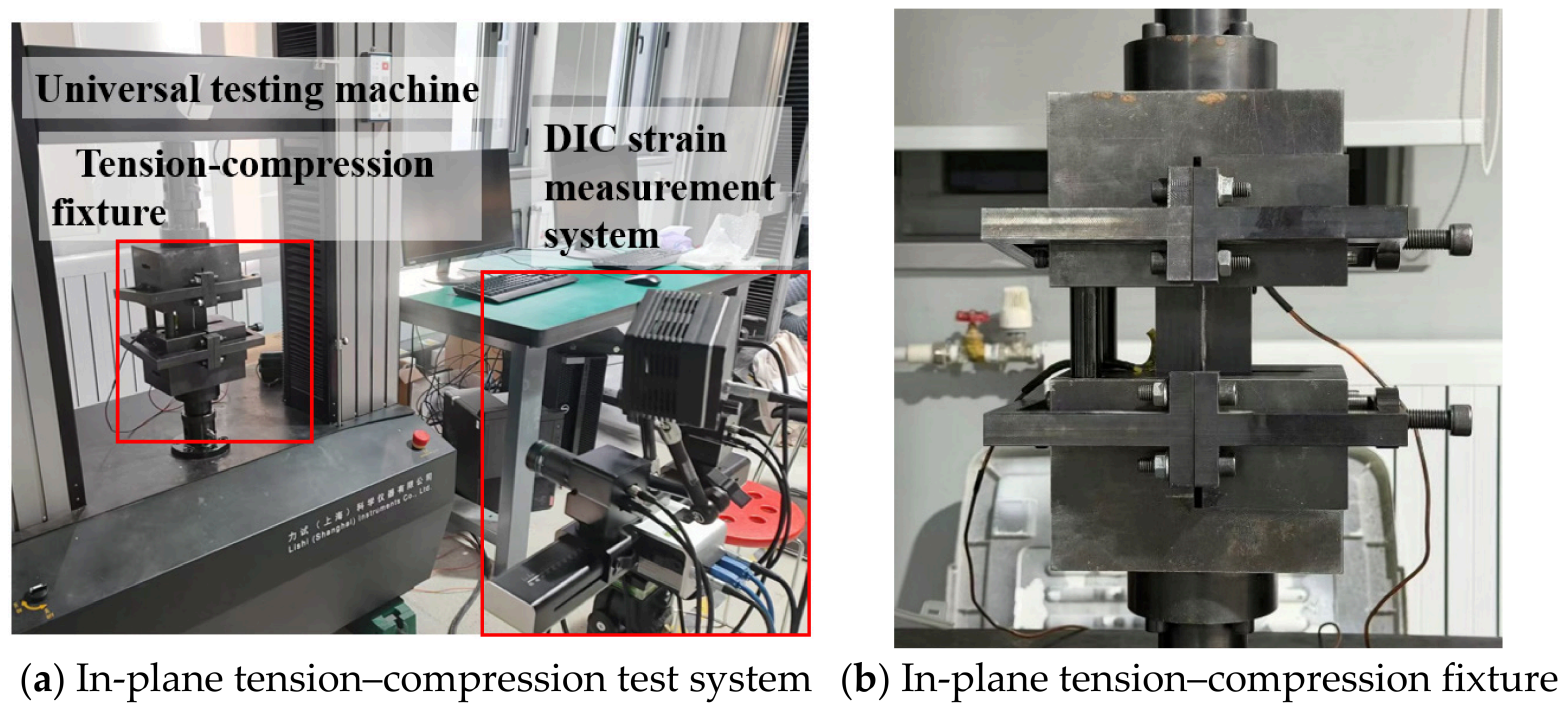
In-plane tension–compression test setup.

The in-plane tension–compression test setup is shown in Figure 5 and mainly consists of three parts: the in-plane tension–compression fixture, the LE5105 LISHI electronic universal testing machine (Lishi Scientific Instruments Co., Ltd., Shanghai, China), and the XTDIC three-dimensional full-field strain measurement system. The in-plane tension–compression fixture was mounted on the electronic universal testing machine, which was used to apply the axial displacement load. The displacement control accuracy was 0.1 mm, and the maximum load capacity was 100 kN. The in-plane tension–compression fixture was used to provide a precisely controllable lateral clamping force on both sides of the specimen parallel section, while the XTDIC system was used to acquire the strain distribution on the exposed thickness-edge surface of the specimen in real time during deformation. The axial strain used for constructing the tension–compression stress–strain curves was obtained from the strain component along the loading direction within this exposed edge region. The system consisted of two 12-megapixel cameras. The camera frame rate was 30 fps, while the image acquisition frequency used in the present test was set to 4 frames per second. The strain measurement range was 0.005–2000%, and the displacement measurement accuracy was less than 0.005 mm. Before the test, the DIC system was calibrated according to the standard calibration procedure of the equipment.

The experimental procedure was as follows. First, the specimen was mounted in the fixture and fixed using screws, and the lateral clamping force was adjusted to the preset value using the side nuts. Subsequently, the electronic universal testing machine drove the fixture to pre-stretch the specimen. After the preset strain value was reached, reverse-compression loading was applied until the end of the test.

### 3.3. Finite Element Model

**Figure 6:**
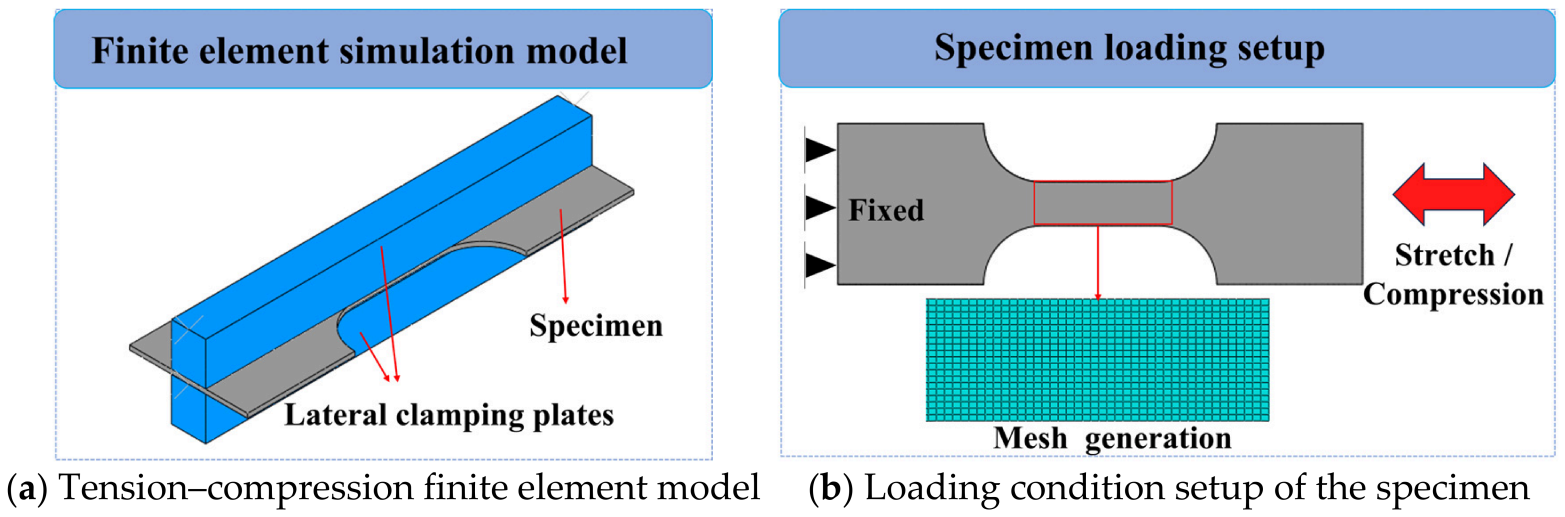
Finite element simulation model and boundary conditions.

To analyze the effect of clamping force on the compressive instability behavior of the specimen under ideal lateral constraint conditions, a finite element simulation model for the tension–compression testing process of the three materials was established using Abaqus software, based on the principle of the in-plane tension–compression test and the measured mechanical parameters of Q890 high-strength steel, 2A14 aluminum alloy, and 304 stainless steel. The finite element model mainly consisted of the specimen and the clamping plates on both sides, as shown in Figure 6.

The specimen was modeled as a deformable body and discretized using C3D8R eight-node linear hexahedral reduced-integration elements. Two element layers were arranged through the thickness direction, and the total number of elements in the specimen model was 5092. The mesh size in the non-parallel section was set to 2 mm × 2 mm. To more accurately capture local buckling deformation in the parallel section, a refined mesh of 1 mm × 1 mm was used, as shown in Figure 6b. The plastic behavior of the specimen material was described using the Chaboche combined hardening model. This model was adopted to account for the loading-path reversal, transient softening, and Bauschinger effect during reverse compression after pre-tension. The two clamping plates were modeled as three-dimensional discrete rigid bodies using R3D4 quadrilateral shell elements. The boundary conditions were defined as follows: one end of the specimen was fixed, while an axial displacement load was applied to the other end. The clamping plates on both sides were used to simulate the lateral clamping structure in the experiment. One clamping plate was fixed, while the other moved with the specimen, reflecting the combined clamping state of the fixed plate and the follower plate in the actual experiment. The clamping load was applied to the outer surface of the clamping plate and transmitted to the specimen surface through the contact between the clamping plate and the specimen. The contact interfaces between the two clamping plates and the specimen were both defined as surface-to-surface contact. Based on the experimental contact condition and commonly reported friction-coefficient ranges for similar metal contact pairs, an equivalent friction coefficient of *μ* = 0.1 was adopted in the finite element model.

## 4. Results and Discussion

Based on the theoretical model established above, this section takes three typical sheet metals, namely Q890 high-strength steel, 2A14 aluminum alloy, and 304 stainless steel, as the research objects. Finite element simulation analysis and experimental validation of the critical clamping force are carried out sequentially to systematically reveal the mechanism of specimen buckling and the effect of clamping force in in-plane tension–compression tests.

### 4.1. Effects of Material Parameters on the Theoretical Critical Pressure

To analyze the influence of material parameters on the critical pressure for compressive instability of in-plane tension–compression specimens, the strength coefficient K, hardening exponent n, and the actual parameters of the three materials were separately substituted into the theoretical model for calculation. In this section, Mode I was selected for critical pressure analysis in the parametric study. Mode I corresponds to low-order single-wave buckling, which is the basic instability form most likely to occur in the early stage of compression.

**Figure 7:**
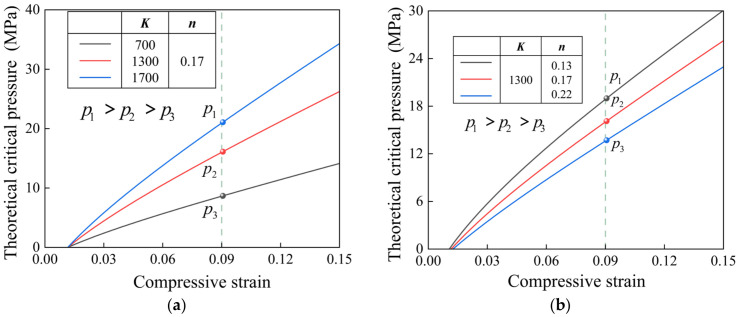
Effects of strength coefficient and hardening exponent on the theoretical critical pressure. (**a**) Effect of material strength coefficient *K* on the critical pressure. (**b**) Effect of material hardening exponent *n* on the critical pressure.

Figure 7 shows the effects of the strength coefficient *K* and hardening exponent n on the theoretical critical pressure. When the hardening exponent was n = 0.17, the theoretical critical pressure increased with increasing strength coefficient *K*, as shown in Figure 7a. Under the same compressive strain, a larger *K* value corresponds to a higher material flow stress level; therefore, a higher lateral support pressure is required to suppress compressive instability. When the strength coefficient was *K* = 1300 MPa, the theoretical critical pressure decreased with increasing hardening exponent n, as shown in Figure 7b. This indicates that, in the theoretical model proposed in this study, an increase in the hardening exponent can enhance the stable deformation capacity of the material during compression, thereby reducing the critical support pressure required to suppress instability.

**Figure 8:**
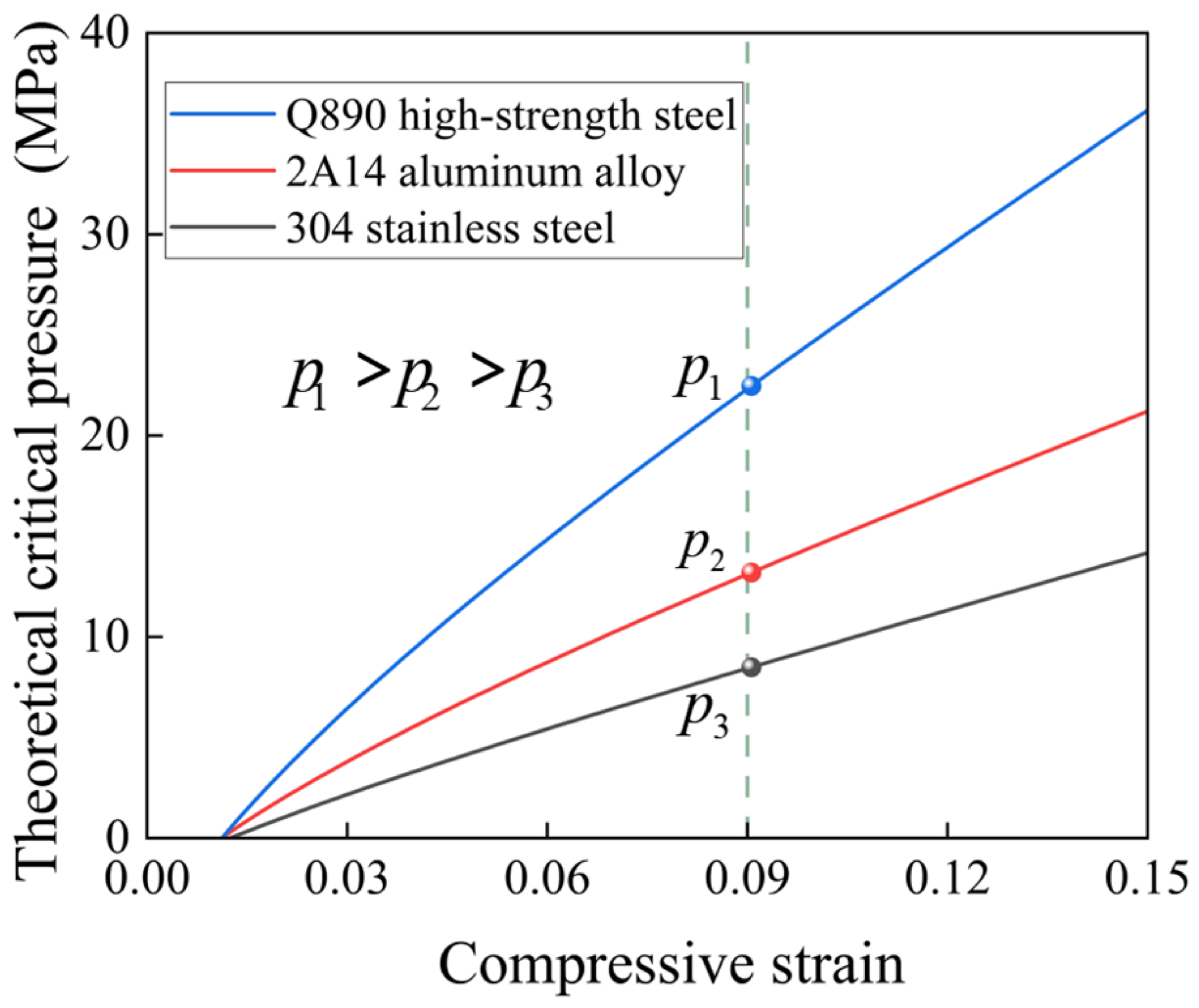
Effect of different materials on the theoretical critical pressure.

The material parameters of Q890 high-strength steel, 2A14 aluminum alloy, and 304 stainless steel were substituted into the model to obtain the variation in theoretical critical pressure for different materials, as shown in Figure 8. Under the same compressive strain, the theoretical critical pressure was the highest for Q890 high-strength steel, intermediate for 2A14 aluminum alloy, and the lowest for 304 stainless steel. This is because Q890 high-strength steel has a higher strength coefficient and therefore a higher corresponding flow stress level during compression deformation, requiring the highest theoretical lateral support pressure. Although 304 stainless steel has a relatively high hardening exponent, its strength coefficient is lower, resulting in the lowest theoretical critical pressure.

In summary, the theoretical model established in this study can reflect the influence of the material strength coefficient and hardening exponent on the critical pressure. The theoretical critical pressure increases with increasing strength coefficient *K* and decreases with increasing hardening exponent *n*. The differences in theoretical critical pressure among different materials indicate that the lateral supporting level required in in-plane tension–compression tests is closely related to the mechanical properties of the material.

### 4.2. Compressive Instability Analysis of Q890 High-Strength Steel Under Different Clamping Forces

To investigate the effect of clamping force on the compressive instability behavior of in-plane tension–compression specimens under ideal lateral constraint conditions, finite element simulations were conducted for Q890 high-strength steel specimens with a pre-strain of 0.03. By analyzing the true stress–strain curves and compressive instability strains of the specimens under different clamping forces, the influence of lateral constraint on compressive stability was examined.

**Figure 9:**
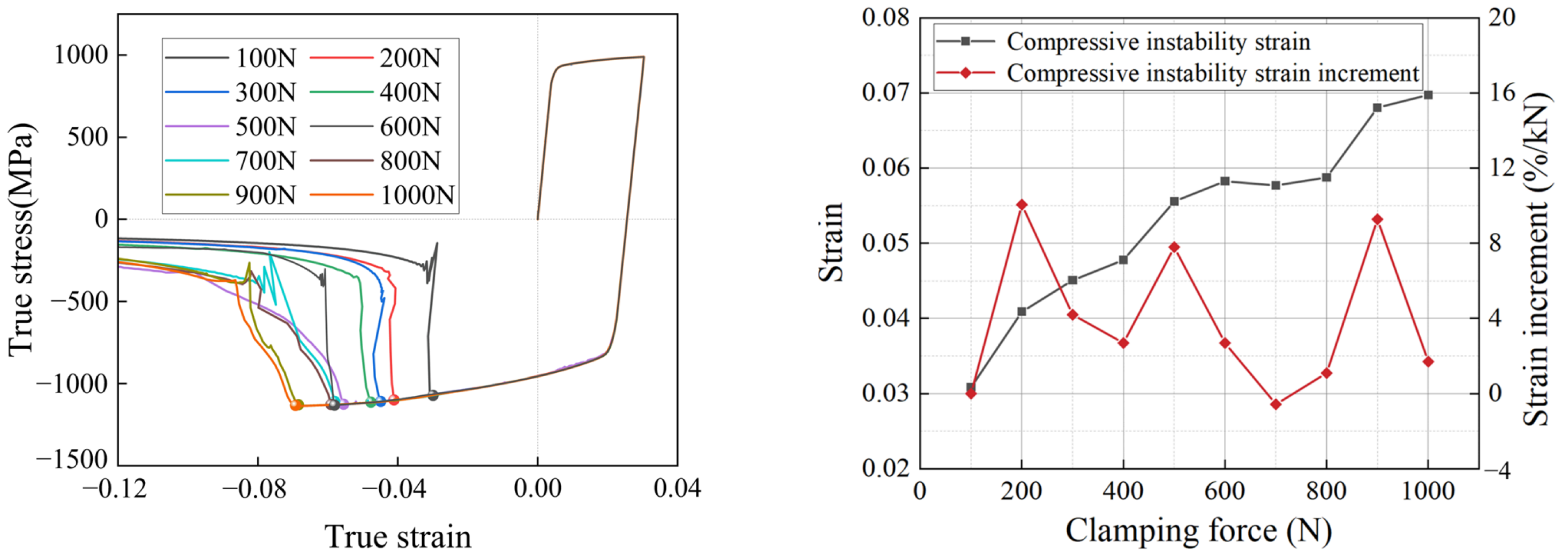
Stress–strain curves and variation in compressive instability strain of Q890 high-strength steel under different clamping forces.

Figure 9a shows the true stress–strain curves of Q890 high-strength steel under different clamping forces. As can be seen from the figure, the compressive strain attainable by the specimen gradually increases with increasing clamping force. However, this increase is not uniform or continuous, but exhibits clear staged characteristics. In the range of 100–400 N, the specimen undergoes instability at an early stage. In the range of 500–800 N, the instability point continues to shift backward but shows certain fluctuations. In the range of 900–1000 N, the specimen can achieve a significantly larger stable compressive strain.

To quantify the improvement in compressive stability within different clamping force intervals, the compressive instability strain corresponding to each clamping force was extracted. The increment of compressive instability strain $\Delta \varepsilon_{i}$ was defined as the ratio of the difference between the absolute values of the compressive instability strains under two adjacent clamping forces to the increment in clamping force, with a unit of % kN^−1^, i.e.,

(21) $$\Delta \varepsilon_{crii}=\frac{\left(\left|\varepsilon_{crit,i}\right|-\left|\varepsilon_{crit,i-1}\right|\right)}{(F_{i}-F_{i-1})/1000}\times 100$$

where $\varepsilon_{i}$ and $\varepsilon_{i-1}$ are the compressive instability strains corresponding to the i-th and (i − 1)-th clamping force conditions, respectively; $F_{i}$ and $F_{i-1}$ are the corresponding clamping forces, respectively, with a unit of N.

As shown in Figure 9b, the compressive instability strain continuously increases with increasing clamping force, but its rate of change exhibits staged fluctuations. In the range of 100–400 N, the compressive instability strain undergoes the first-stage increase. In the range of 500–800 N, it undergoes the second-stage increase and enters a relatively gradual region. In the range of 900–1000 N, a third-stage significant jump occurs, after which the increment tends to decrease again. This result indicates that the improvement in the compressive stability of Q890 high-strength steel by the clamping force is not linear, but exhibits a staged transition characteristic.

### 4.3. Contact-State Evolution and Instability-Mode Transition of Q890 High-Strength Steel Under Different Clamping Forces

**Figure 10:**
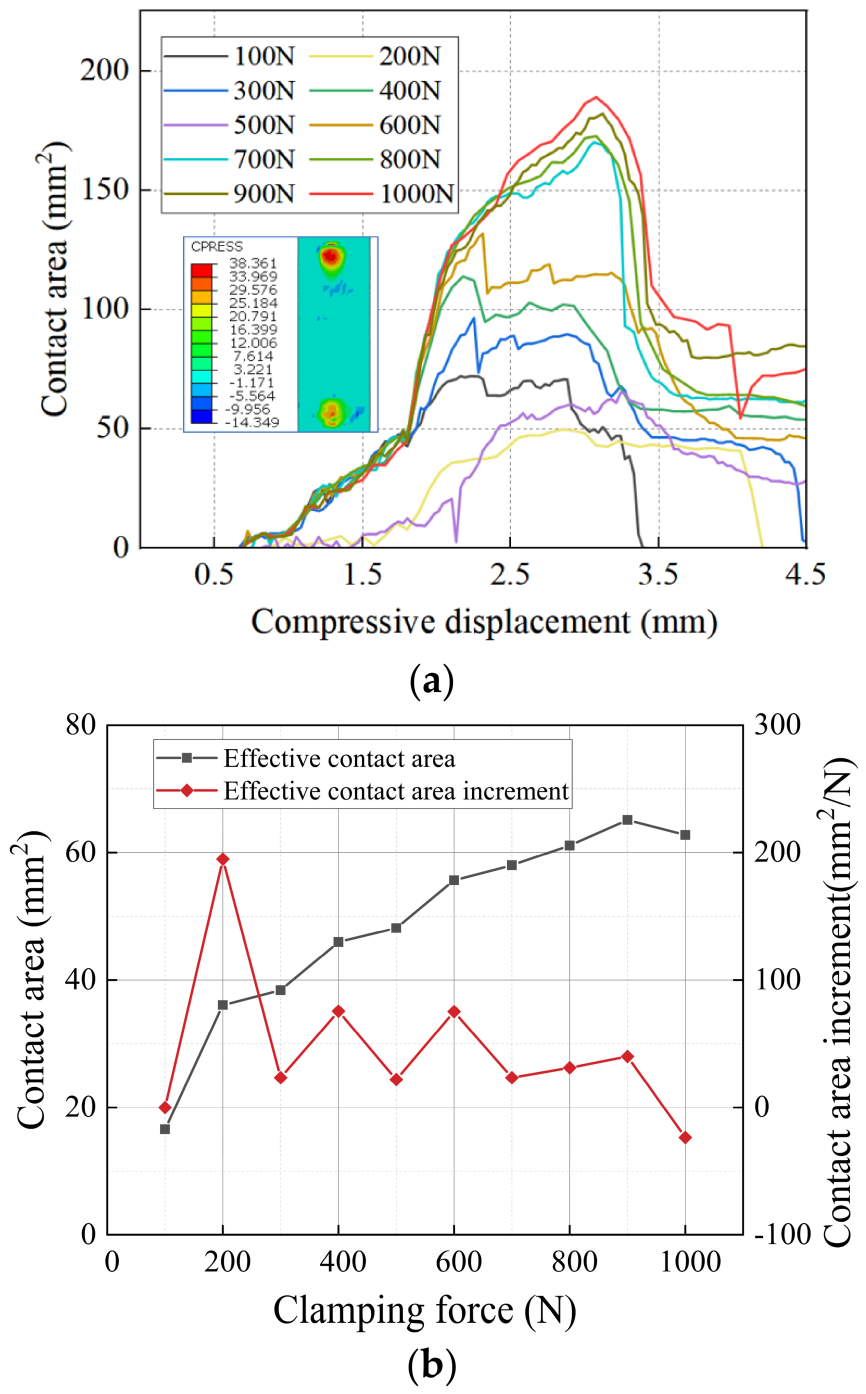

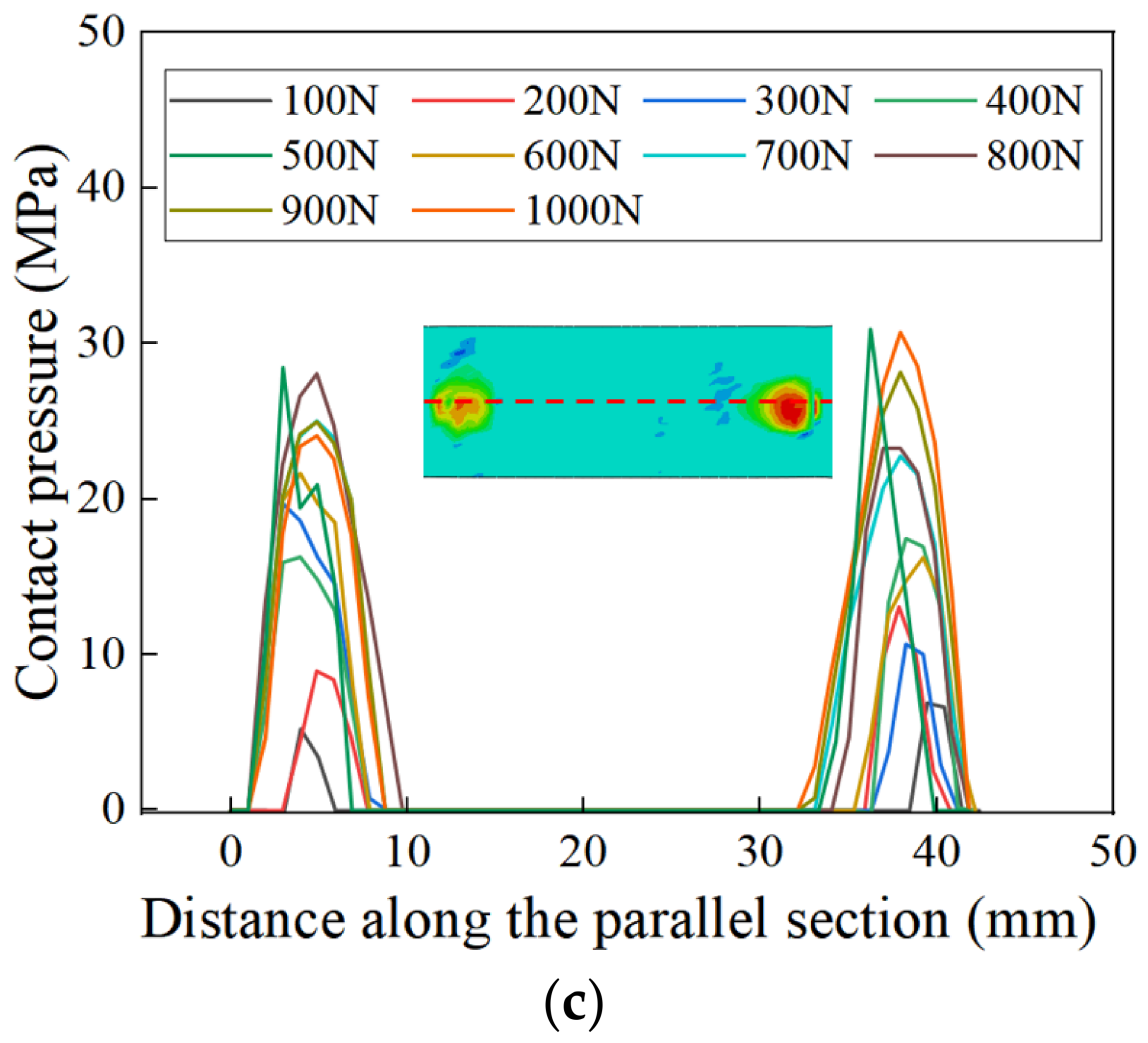
Contact-state variation in Q890 high-strength steel under different clamping forces. (**a**) Compressive displacement–contact area curves. (**b**) Effective contact area and its increment. (**c**) Contact pressure distribution along the centerline of the parallel section.

To further reveal the mechanism by which the clamping force affects compressive stability, the contact state between the specimen and the clamping plates under different clamping forces was systematically analyzed. Figure 10a shows the variation in contact area between the specimen parallel section and the two lateral clamping plates with compressive displacement. Under different clamping forces, the contact area first increases and then decreases as the compressive displacement increases. In the range of 200–600 N, the peak contact area is relatively low, but its increase with increasing clamping force is relatively large. In the range of 700–1000 N, the peak contact area is relatively high, but the increase becomes less pronounced as the contact area increases. It is worth noting that the contact area does not increase strictly monotonically with increasing clamping force. For example, the peak contact areas at clamping forces of 200 N and 600 N are lower than those at 100 N and 500 N, respectively. This non-monotonic variation indicates that the total contact area is not determined solely by the magnitude of the lateral clamping force. During compression, the local buckling morphology, contact opening and closure, local gap variation between the specimen and the clamping plates, and redistribution of contact pressure may also affect the instantaneous contact area. Therefore, the total contact area only reflects the overall geometric contact state and cannot be directly regarded as the effective lateral support acting on the specimen. Nevertheless, increasing the clamping force generally enhances the contact between the specimen and the clamping plates and helps maintain a more stable specimen state during compression.

For suppressing compressive instability, the mechanically effective region is the high-pressure contact region that can provide sufficient local support. To further investigate the actual effective support formed during the stable compression stage under different clamping forces, the effective contact regions at the same compressive strain under different clamping forces were extracted and compared. In this study, the effective contact-pressure threshold for Q890 high-strength steel was determined as 0.7 MPa, which corresponds to approximately 1/30 of the peak contact pressure obtained from the finite element simulations. Therefore, the region with a contact pressure higher than 0.7 MPa was defined as the effective contact region. This criterion was used to exclude weak or incidental contact regions with very low normal pressure and to identify the high-pressure contact region that can provide mechanically effective lateral support.

To quantitatively characterize the variation in the effective contact area with increasing clamping force, the increment in effective contact area, $\Delta S_{i}$ was defined as the ratio of the difference in effective contact area between two adjacent clamping force conditions to the corresponding clamping force increment:

(22) $$\Delta S_{i}=\frac{S_{i}-S_{i-1}}{F_{i}-F_{i-1}}\times 100$$

where $S_{i}$ and $S_{i-1}$ are the effective contact areas under the i and i−1 clamping force conditions, respectively, with a unit of mm^2^; *F_i_* and *F_i_*_−1_ are the corresponding clamping forces, with a unit of N; and $\Delta S_{i}$ represents the average variation in effective contact area caused by a unit increment in clamping force, with a unit of mm^2^/N. The factor of 100 in Equation (22) is used only to amplify the numerical values, thereby facilitating comparison among different clamping force intervals.

Figure 10b shows the effective contact area under different clamping forces. As the clamping force increases, the effective contact area shows an overall increasing trend. In the range of 100–500 N, the effective contact area is generally small, but the increase is relatively large. In the range of 600–1000 N, the effective contact area continues to increase, but the increment decreases. This result indicates that the effective contact area can better reflect the effective lateral support capacity acting on the specimen during compression than the total contact area.

Figure 10c shows the contact pressure distribution extracted along the centerline of the parallel section at the same compressive strain under different clamping forces. It can be seen that the contact pressure is mainly concentrated at both ends of the parallel section, while almost no contact pressure exists in the middle region, indicating that the lateral constraint effect has an obvious localized characteristic. As the clamping force increases from 100 to 400 N to 500–800 N and further to 900–1000 N, both the peak pressure and the acting range of the high-pressure regions at both ends gradually expand. In particular, in the transition range of 500–800 N, a sudden increase in the local pressure peak, asymmetric distribution, and enhanced local concentration can be observed. This indicates that the improvement in compressive stability depends not only on the magnitude of the contact area, but is also closely related to whether sufficiently strong local pressure support is formed in key regions. The effect of increasing the clamping force is reflected not only in increased contact, but also in the enhancement of effective support capacity.

**Figure 11:**
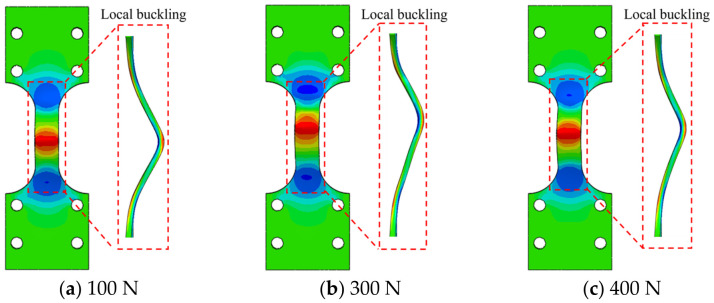

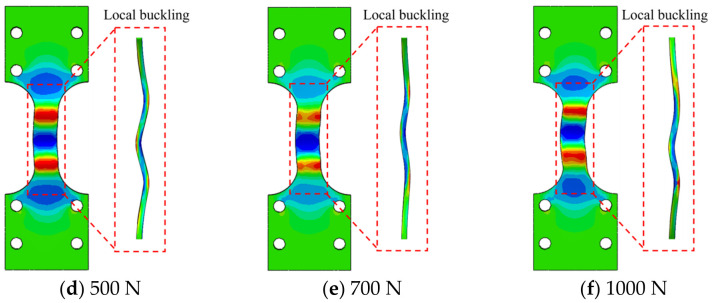
Buckling morphologies of Q890 high-strength steel under different clamping forces.

The contact-state distributions and instability profiles under different clamping forces are shown in Figure 11. When the clamping force is 100 N, 300 N, and 400 N, the specimen exhibits single-wave buckling. When the clamping force is increased to 500 N, the instability mode switches to double-wave buckling, and this double-wave buckling is maintained at 700 N and 1000 N. The mode change is highly consistent with the evolution of the contact state. Under low clamping forces, the high-pressure contact region is relatively small and the local support is insufficient, making single-wave buckling more likely to form. As the clamping force increases, the area of the high-pressure contact region expands, the local pressure support at both ends is enhanced, and the instability mode changes to a more stable double-wave buckling.

In summary, the staged improvement in the compressive stability of Q890 high-strength steel is closely associated with the expansion of the high-pressure contact region and the enhancement of local support capacity. These changes correspond to the transition of the instability mode from single-wave to double-wave buckling.

### 4.4. Experimental Analysis of the Critical Clamping Force Interval for Q890 High-Strength Steel

To verify the effect of lateral clamping force on the reverse-compression instability behavior of pre-tensioned sheets, in-plane tension–compression tests were conducted on Q890 high-strength steel under different clamping forces. In this study, the onset of com-pressive instability was identified based on the variation in the compression stress–strain curve. When local instability begins, the tangent slope of the compression curve changes from positive to negative, accompanied by local fluctuation or local upward turning of the stress–strain curve. In addition, the DIC images were used as auxiliary evidence to confirm the occurrence of local bending or out-of-plane deformation, thereby supporting the identification of the instability onset.

**Figure 12:**
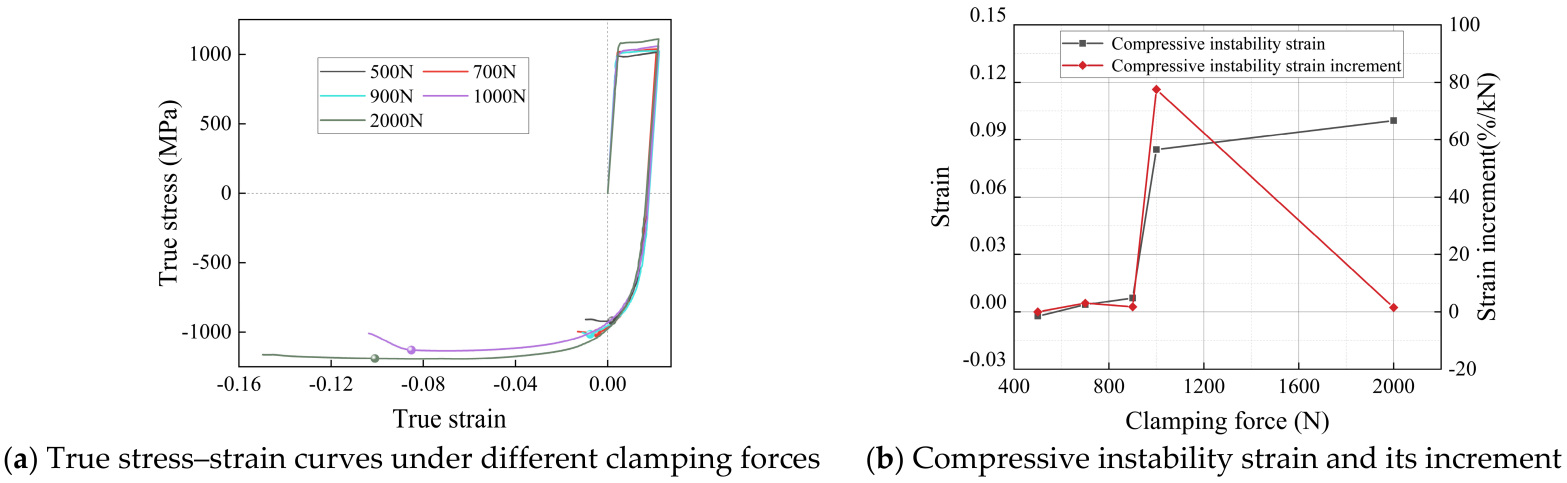
Stress–strain curves and variation in compressive instability strain of Q890 high-strength steel under different clamping forces.

Figure 12 shows the true stress–strain curves of Q890 high-strength steel under different clamping forces at a pre-tensile strain of 0.02. In the clamping force range of 500–900 N, the stable compression segment is relatively short, and instability occurs within a small compressive strain range. As quantitatively shown in Figure 12b, the compressive instability strains under clamping forces of 500 N, 700 N, and 900 N are 0.006, 0.001, and 0, respectively, all below 0.01. This indicates that increasing the clamping force within this range has a very limited effect on improving compressive stability. However, when the clamping force is increased from 900 N to 1000 N, the stable compression segment is significantly extended, and the compressive instability strain jumps to 0.085. The corresponding strain increment reaches its peak at this point, with a value of 77.5%, indicating that the compressive stability of Q890 high-strength steel undergoes a sharp increase within this interval. When the clamping force is further increased to 2000 N, the compressive instability strain only increases from 0.085 to 0.095, with an increment of 0.01, while the strain increment decreases to 1.5%. This indicates that further increasing the clamping force has an extremely limited effect.

In summary, the compressive stability of Q890 high-strength steel does not increase continuously and linearly with increasing clamping force. Instead, a relatively clear critical interval exists near 900–1000 N. Below this interval, the compressive instability strain remains at a low level; above this interval, the stable compression capacity is significantly enhanced; and further increasing the clamping force produces no obvious improvement.

**Figure 13:**
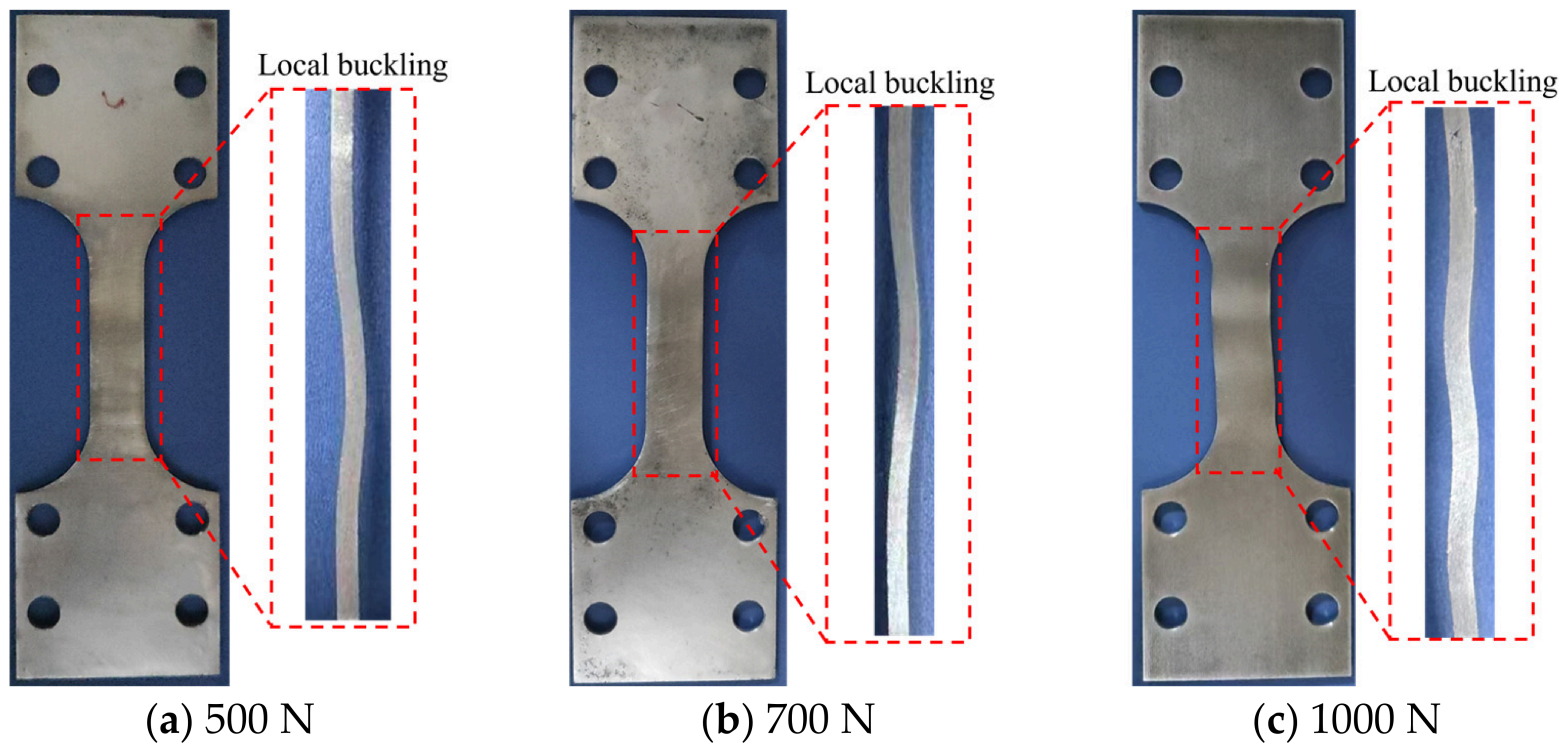
Buckling morphologies of Q890 high-strength steel under different clamping forces.

A comparison of the instability morphologies of the specimens under different clamping forces is shown in Figure 13. Clear differences in instability morphology can be observed under different clamping forces. When the clamping force is below 900 N, the compressive instability strain of the specimen is lower than 0.02, and the specimen exhibits single-wave buckling. When the clamping force is increased to 1000 N, the compressive instability strain increases to 0.085, and the instability mode switches from single-wave to double-wave. This experimental result is qualitatively consistent with the trend revealed by the simulation in Section 4.3, namely the expansion of the high-pressure contact region and the enhancement of local support with increasing clamping force. However, the transition clamping force observed in the experiment is higher than that predicted by the idealized FE model. This discrepancy can be mainly attributed to the difference between the idealized contact/boundary conditions in the FE model and the actual fixture condition in the experiment. In the actual test, fixture compliance, bolt-tightening deviation, slight misalignment, surface roughness, frictional resistance, and nonuniform force transmission may reduce the effective lateral support acting on the specimen. Therefore, a higher nominal clamping force is required in the experiment to achieve the same level of effective support. As a result, the improvement in stability is more concentrated in the experiment, occurring mainly in the range of 900–1000 N, and the staged characteristic is more pronounced.

### 4.5. Correction of the Critical Clamping Force Model

The theoretical model established above is derived under ideal uniform boundary conditions and therefore provides a critical support-pressure baseline rather than the actual clamping force required in experiments. In actual in-plane tension–compression tests, the contact state between the specimen and the clamping plates is localized and nonuniform. Therefore, the actual clamping force required to suppress wrinkling depends not only on the theoretical support pressure, but also on the effective contact region through which the normal force is transmitted.

The parallel section length of the in-plane tension–compression specimen is 40 mm. Based on the instability morphologies observed in the experiments, the low-order and high-order instability modes were respectively equivalent to instability forms with different wave numbers within the parallel section. According to the correspondence between the parallel section length and the instability wave number, the wavelengths corresponding to single-wave and double-wave buckling were taken as λ=20 and λ=10, respectively, and were substituted into the theoretical critical support pressure model for calculation.

**Figure 14:**
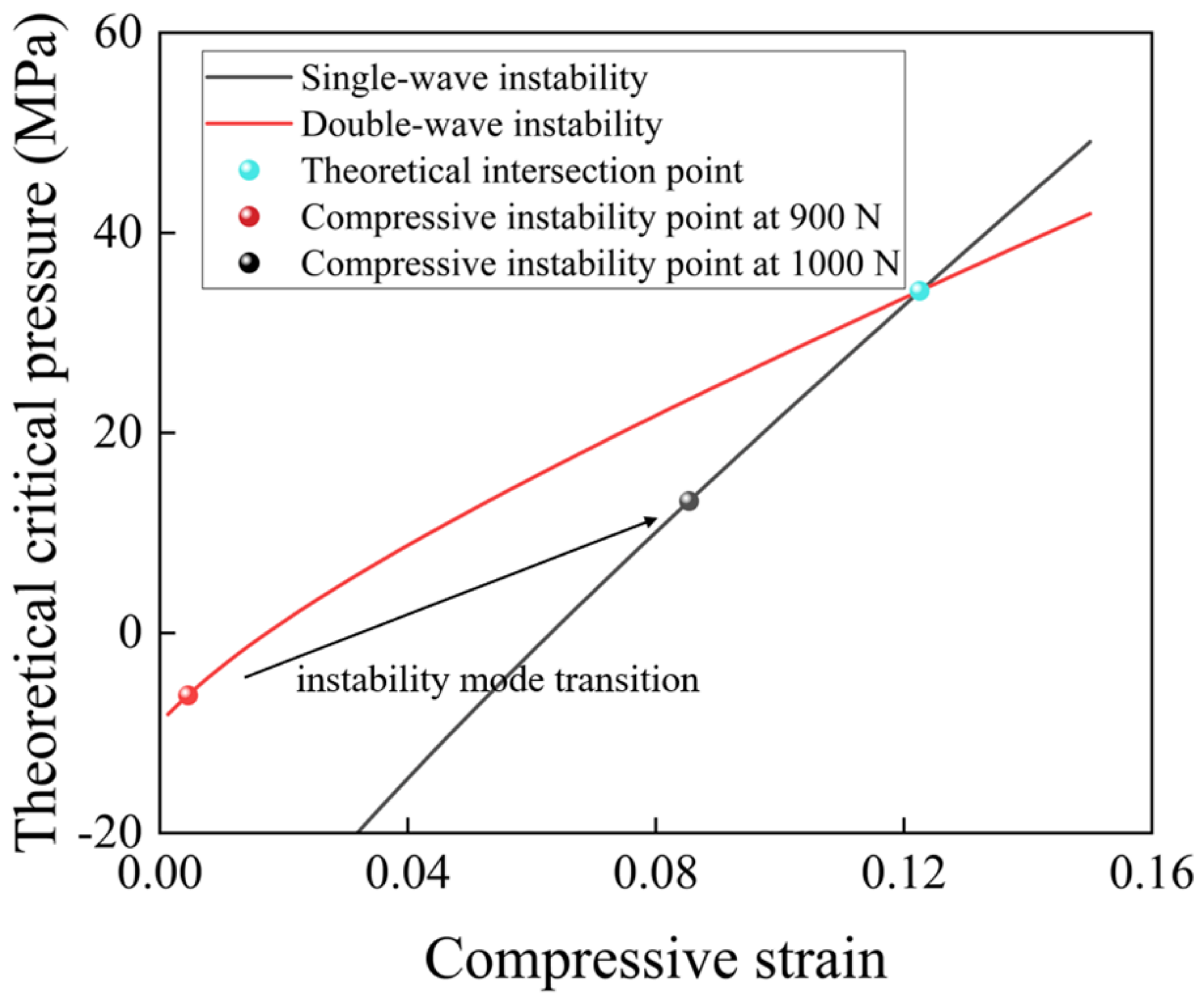
Comparison between the theoretical critical support pressure curves and experimental instability points of Q890 high-strength steel.

The comparison between the theoretical critical pressure curves and the experimental instability points for Q890 high-strength steel is shown in Figure 14. At 1000 N, the specimen undergoes a mode transition from single-wave to double-wave buckling, and the instability strain increases substantially compared with that at 900 N. This indicates that the mode transition of the actual specimen does not occur at the theoretical intersection point, but appears before reaching the ideal intersection.

Therefore, a boundary correction coefficient η is introduced in this study to correct the theoretical model. The experimental state corresponding to the mode transition of Q890 high-strength steel was used as the calibration reference. The compressive instability strain was taken as $\varepsilon_{ref}=0.085$ corresponding to the instability strain at 1000 N. The critical pressure predicted by the theoretical model at this strain is denoted as $p_{th}$:


(23) $$p_{th}=p_{th}(\varepsilon_{ref},K,n)$$

The experimental mode-transition clamping force of Q890 high-strength steel, 1000 N, was taken as the reference value, namely,


(24) $$F_{\exp}=1000N$$

Thus, the corrected critical clamping force can be expressed as:

(25) $$F_{pre}^{corr}=\eta \cdot p_{th}\cdot A_{par}$$

where $A_{par}$ is the area of the specimen parallel section. The value of *η* obtained from the Q890 calibration data is 0.127. Thereafter, this boundary correction coefficient was substituted into the theoretical models of the other materials to predict the critical clamping force corresponding to their mode transitions.

### 4.6. Experimental Results of 2A14 Aluminum Alloy and 304 Stainless Steel

**Figure 15:**
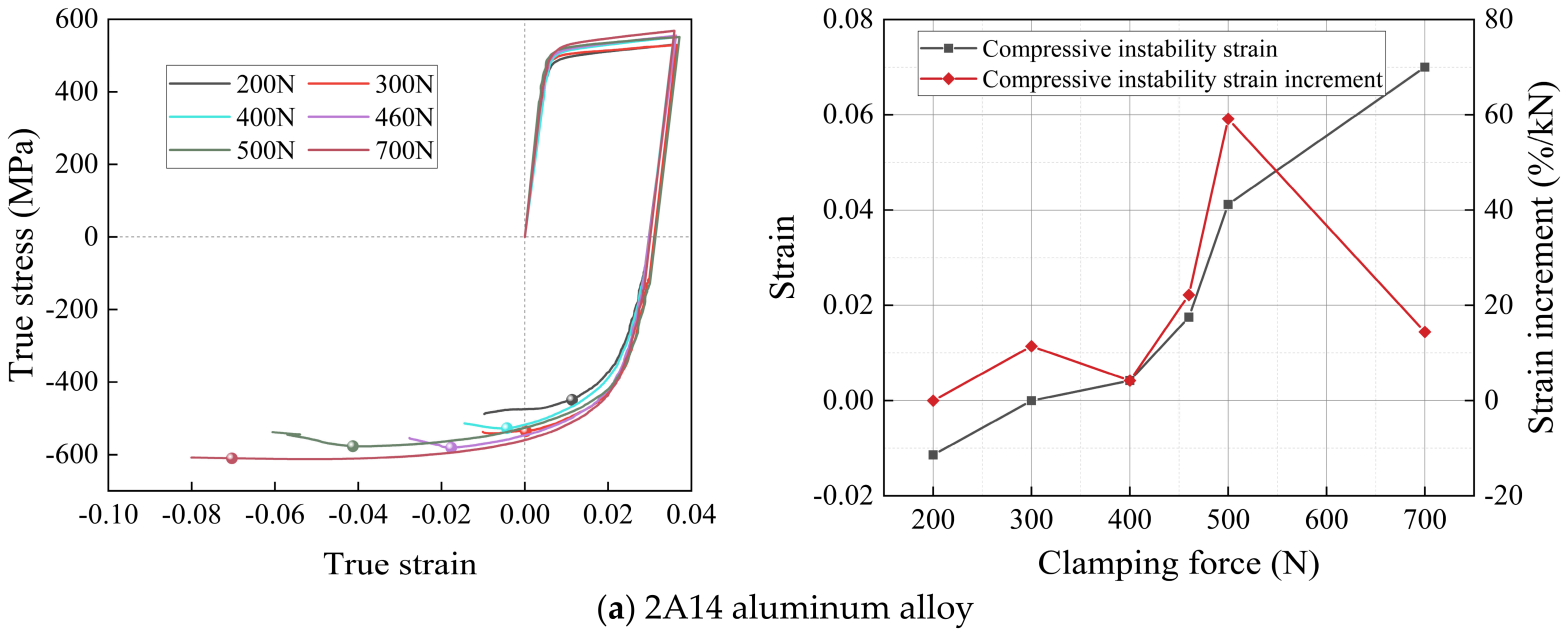

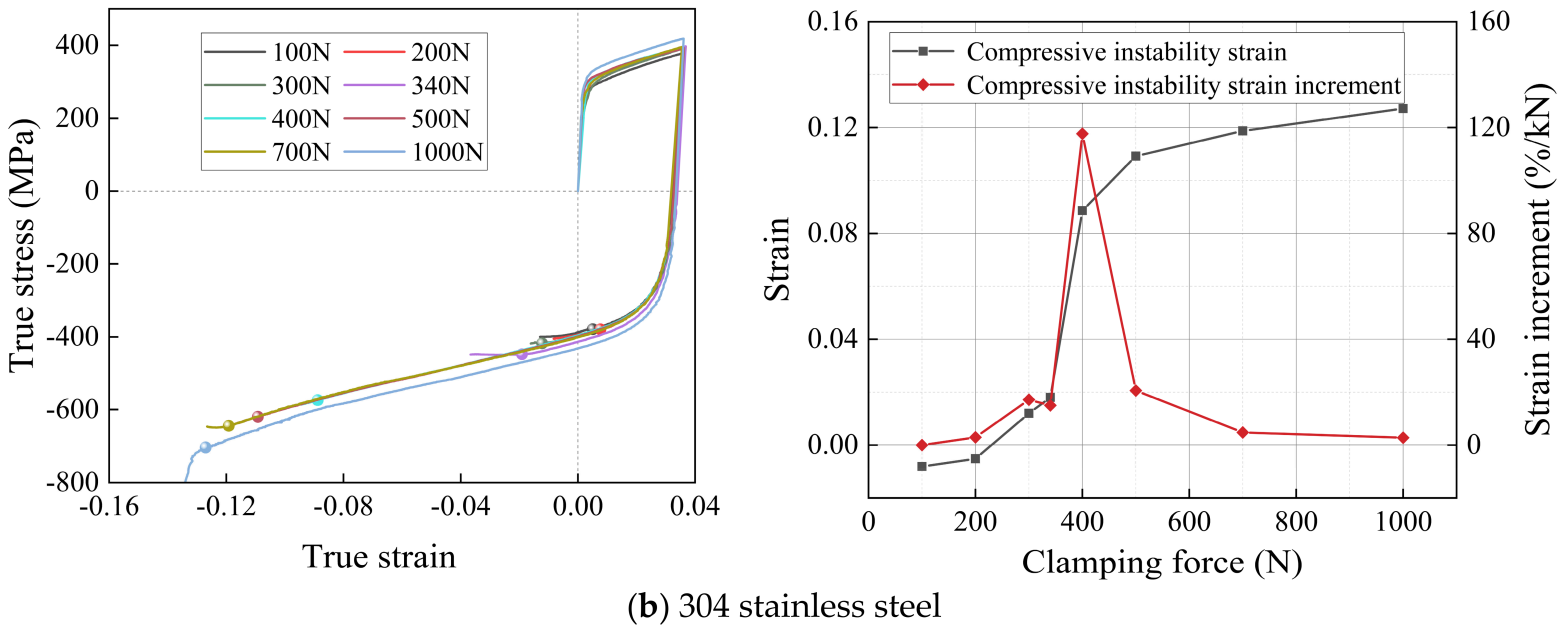
True stress–strain curves and variation in compressive instability strain of 2A14 aluminum alloy and 304 stainless steel.

To further examine the variation in compressive stability of different materials under clamping force, in-plane tension–compression tests were conducted on 2A14 aluminum alloy and 304 stainless steel under different clamping forces. The obtained true stress–strain curves and variations in compressive instability strain are shown in Figure 15.

Figure 15a shows the true stress–strain curves and variation in compressive instability strain of 2A14 aluminum alloy. It can be seen that the compressive instability strains of 2A14 aluminum alloy are relatively low in the clamping force range of 200–400 N, with values of −0.011, 0, and 0.004, respectively. This indicates that the specimen undergoes compressive instability at an early stage under low clamping forces. When the clamping force increases to 460 N and 500 N, the compressive instability strain increases to 0.018 and 0.041, respectively, indicating a clear improvement in stable compression capacity. When the clamping force is further increased to 700 N, the compressive instability strain reaches 0.070, while the increment decreases. Therefore, the compressive stability of 2A14 aluminum alloy mainly improves within the range of 460–500 N.

Figure 15b shows the true stress–strain curves and variation in compressive instability strain of 304 stainless steel. It can be seen that the compressive instability strain of 304 stainless steel is relatively low in the clamping force range of 100–340 N, increasing from −0.009 to 0.017, and the improvement in stable compression capacity is limited. When the clamping force increases to 400 N, the compressive instability strain increases to 0.088, showing a significant improvement. When the clamping force is further increased to 500 N, 700 N, and 1000 N, the compressive instability strains are 0.108, 0.118, and 0.126, respectively, with gradually decreasing increments. This indicates that the compressive stability of 304 stainless steel mainly undergoes a jump within the range of 340–400 N.

### 4.7. Predicted Critical Clamping Forces and Experimental Validation for Different Materials

**Figure 16:**
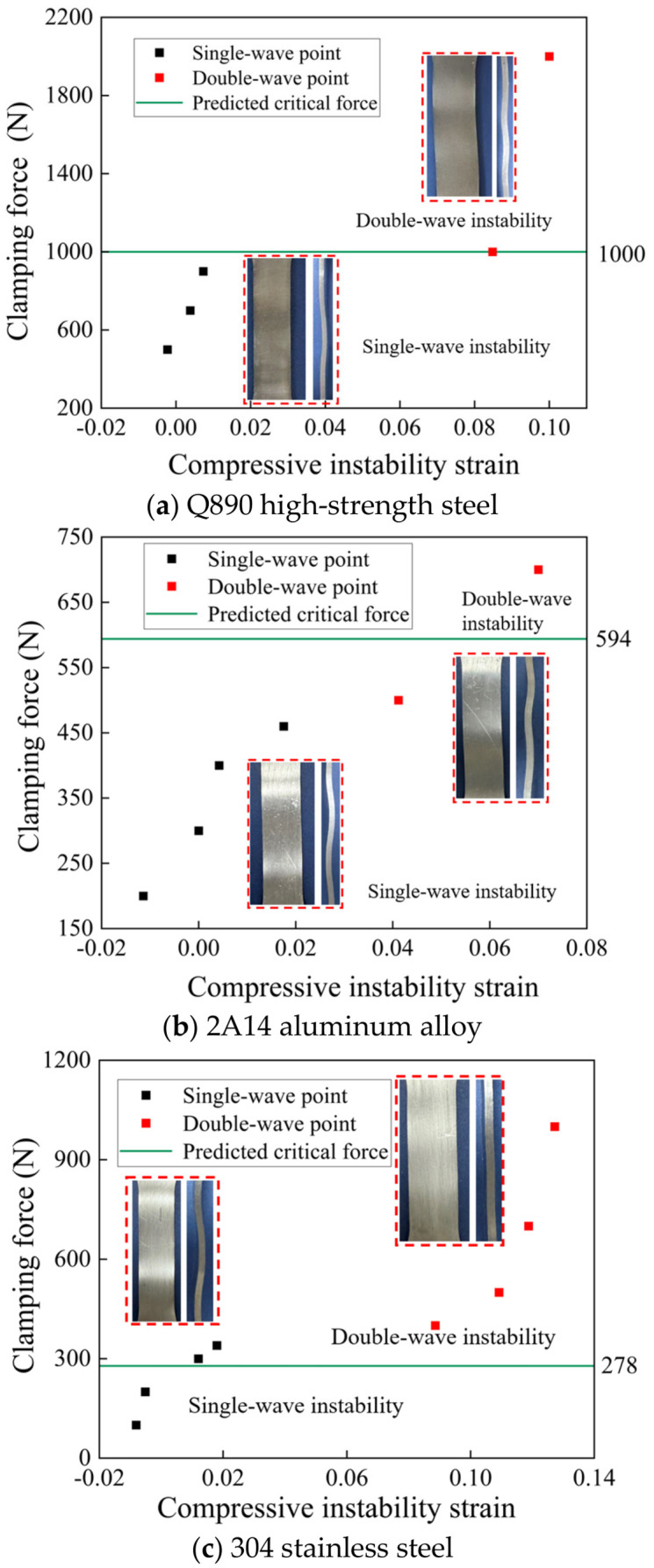
Comparison between corrected model predictions and experimental critical clamping force intervals for different materials.

To verify the applicability of the corrected model, the experimental results of 304 stainless steel and 2A14 aluminum alloy shown in Figure 16 were used as references. Q890 high-strength steel was used as the calibration reference for determining the boundary correction coefficient. The boundary correction coefficient calibrated from Q890 high-strength steel was substituted into the theoretical models of the two materials, and the predicted critical clamping forces were compared with the clamping force intervals corresponding to the experimentally observed mode transitions.

Figure 16b shows the comparison between the corrected model prediction and the experimental critical clamping force interval for 2A14 aluminum alloy. By substituting the material parameters of 2A14 aluminum alloy into the corrected model, the predicted critical clamping force was obtained as 594 N. In the clamping force range of 200–460 N, 2A14 aluminum alloy exhibits single-wave buckling and relatively low compressive instability strain. When the clamping force is increased to above 500 N, the specimen gradually switches to double-wave buckling, and the compressive instability strain increases markedly. Taking the experimentally observed transition force of approximately 500 N as the reference, the relative deviation of the predicted value is 18.8%, corresponding to a prediction accuracy of 81.2%.

Figure 16c shows the comparison between the corrected model prediction and the experimental critical clamping force interval for 304 stainless steel. By substituting the material parameters of 304 stainless steel into the corrected model, the predicted critical clamping force was obtained as 278 N. In the clamping force range of 100–340 N, 304 stainless steel exhibits single-wave buckling and relatively low compressive instability strain. When the clamping force is increased to 400 N, the favored instability mode switches to double-wave buckling, and the compressive instability strain increases significantly. Taking the experimentally observed transition force of approximately 400 N as the reference, the relative deviation of the predicted value is 30.5%, corresponding to a prediction accuracy of 69.5%.

The predicted values generally correspond to the clamping force intervals associated with the experimentally observed mode transitions. This indicates that the boundary correction method proposed in this study can effectively incorporate the boundary effects under real lateral constraint conditions while retaining the basic framework of the theoretical model, thereby providing a reference for the preliminary design of clamping force in in-plane tension–compression tests.

### 4.8. Single-Cycle In-Plane Tension–Compression Tests Under Optimized Clamping Forces

Based on the above theoretical prediction, FE simulation, and experimental validation, the optimized clamping force parameters were applied to single-cycle in-plane tension–compression tests of the three materials to further verify their applicability. The final experimental clamping forces were determined as follows: 1000 N for Q890 high-strength steel, 500 N for 2A14 aluminum alloy, and 400 N for 304 stainless steel. The tensile pre-strain was 2–5% for Q890 high-strength steel and 2–9% for 2A14 aluminum alloy and 304 stainless steel.

**Figure 17:**
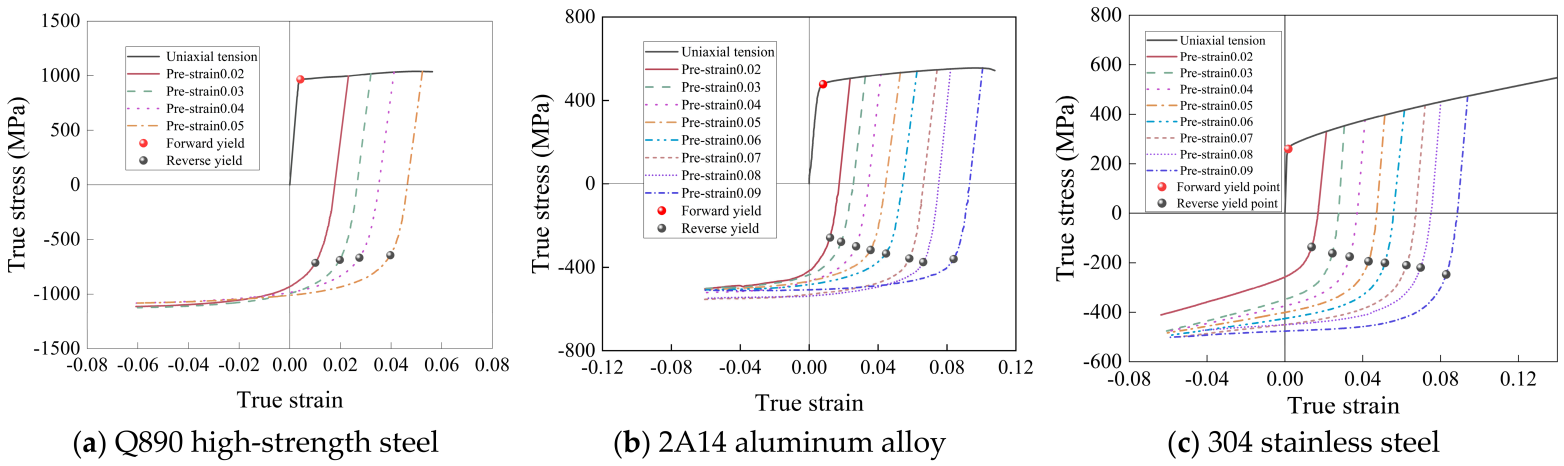
Stress–strain curves of Q890 high-strength steel, 2A14 aluminum alloy, and 304 stainless steel.

Figure 17a–c show the true stress–strain curves of the three materials obtained under the optimized clamping forces. It can be seen that all curves exhibit smooth transitions after loading-path reversal, namely from tension to compression. No abrupt changes in the stress–strain data caused by specimen buckling are observed after yielding in the compression stage. This indicates that the clamping forces determined in this study can effectively suppress compressive instability and accurately reflect the mechanical response of the materials.

## 5. Conclusions

In this study, a theoretical prediction model for the critical clamping force in in-plane tension–compression tests was successfully developed based on the energy method and the Cao–Boyce instability criterion. Combined with finite element simulations and systematic experiments on three typical sheet metals, namely Q890 high-strength steel, 2A14 aluminum alloy, and 304 stainless steel, the compressive instability behavior and the applicability of the model were analyzed in depth. The main conclusions are as follows:

(1) The theoretical model reveals the quantitative relationship between the critical support-pressure baseline and the intrinsic material parameters, which provides a basis for determining the material-dependent clamping force. The critical support pressure increases with the material strength coefficient *K* and decreases with the strain-hardening exponent *n*. Verified by three sheet metals with significantly different mechanical properties, the model can accurately predict the variation trend of the clamping force required for different materials in in-plane tension–compression tests, providing a theoretical basis for material-dependent clamping force design.
(2) To account for the deviation between the ideal model and actual experiments, a boundary-correction-based calibration method was proposed. The theoretical model assumes uniform normal constraint, whereas the actual contact state exhibits localized and nonuniform characteristics. In this study, a boundary correction coefficient was introduced, and the experimental mode-transition state of Q890 high-strength steel was used as the calibration reference to establish the corrected prediction expression for the critical clamping force. Independent validation using 304 stainless steel and 2A14 aluminum alloy showed that the predicted values, 278 N and 594 N, respectively, generally corresponded to the clamping force intervals associated with the experimentally observed mode transitions. This indicates that the correction method can incorporate the influence of real boundary conditions while retaining the basic theoretical framework, showing good engineering applicability among the tested materials.
(3) Stable stress–strain responses were obtained in single-cycle in-plane tension–compression tests under the predicted and optimized clamping forces. The experiments were conducted using the clamping forces determined by the corrected model, namely 1000 N for Q890 high-strength steel, 500 N for 2A14 aluminum alloy, and 400 N for 304 stainless steel. Under these clamping force conditions, the stress–strain responses of the three materials showed smooth transitions during loading-path reversal, without obvious abnormal fluctuations caused by premature buckling in the compression stage. This indicates that the proposed clamping force prediction and correction method can provide suitable clamping force ranges for obtaining stable reverse-loading curves and for characterizing the Bauschinger effect. With simplified assumptions in the theoretical model and an experimentally calibrated boundary correction coefficient, the proposed method can provide theoretical guidance and technical support for the prediction and selection of clamping forces in large-strain in-plane tension–compression tests of different materials.

## Figures and Tables

**Table 1:** Mechanical properties of Q890 high-strength steel, 2A14 aluminum alloy and 304 stainless steel.

Material	*E* (GPa)	Poisson’s Ratio ν	Yield Strength $\sigma_{s}$ (MPa)	Strength Coefficient *K* (MPa)	Hardening Exponent *n*	Fitting Variance *R*^2^
Q890 high-strength steel	215	0.3	950.0	1692.2	0.149	0.9398
2A14 aluminum alloy	80	0.33	420.0	984.3	0.146	0.9327
304 stainless steel	170	0.3	280.0	789.7	0.214	0.9922

## Data Availability

The original contributions presented in this study are included in the article. Further inquiries can be directed to the corresponding authors.
